# Tissue- and liquid-biopsy based NGS profiling in advanced non-small-cell lung cancer in a real-world setting: the IMMINENT study

**DOI:** 10.3389/fonc.2024.1436588

**Published:** 2024-07-09

**Authors:** Marco Sposito, Lorenzo Belluomini, Riccardo Nocini, Jessica Insolda, Ilaria Mariangela Scaglione, Jessica Menis, Michele Simbolo, Antonio Lugini, Federica Buzzacchino, Francesco Verderame, Francesca Spinnato, Giuseppe Aprile, Lorenzo Calvetti, Mario Occhipinti, Daniele Marinelli, Antonello Veccia, Fiorella Lombardo, Hector José Soto Parra, Francesco Ferraù, Clementina Savastano, Camilla Porta, Lorenzo Pradelli, Emilia Sicari, Silvia Castellani, Umberto Malapelle, Silvia Novello, Emilio Bria, Sara Pilotto, Michele Milella

**Affiliations:** ^1^ Section of Innovation Biomedicine – Oncology Area, Department of Engineering for Innovation Medicine (DIMI), University of Verona and University and Hospital Trust/Azienda Ospedaliero-Universitaria Integrata (AOUI), Verona, Italy; ^2^ Otolaryngology-Head and Neck Surgery Department, University of Verona Hospital Trust, Verona, Italy; ^3^ Department of Diagnostics and Public Health, Section of Pathology, University and Hospital Trust of Verona, Verona, Italy; ^4^ Medical Oncology Unit, Azienda Ospedaliera (AO) San Giovanni Addolorata Hospital, Rome, Italy; ^5^ Medical Oncology Unit, San Giuseppe Moscati Hospital, Taranto, Italy; ^6^ Section of Oncology, Azienda Ospedaliera (AO) Ospedali Riuniti “Villa Sofia- V. Cervello”, Palermo, Italy; ^7^ Department of Clinical Oncology, San Bortolo General Hospital, Azienda ULSS8 Berica, Vicenza, Italy; ^8^ Department of Experimental Medicine, Sapienza University, Rome, Italy; ^9^ Medical Oncology Department, Fondazione Istituti di Ricovero e Cura a Carattere Scientifico (IRCCS) Istituto Nazionale Dei Tumori, Milan, Italy; ^10^ Division of Medical Oncology B, Policlinico Umberto I, Rome, Italy; ^11^ Medical Oncology Department, Santa Chiara Hospital, Trento, Italy; ^12^ Lung Unit, Ospedale Pederzoli, Peschiera del Garda, Verona, Italy; ^13^ Medical Oncology, Azienda Ospedaliero Universitaria Policlinico “G. Rodolico-S. Marco”, Catania, Italy; ^14^ Department of Medical Oncology, Unità Operativa Complessa (UOC) Oncologia, Taormina, Italy; ^15^ Medical Oncology Unit, San Giovanni di Dio e Ruggi d’Aragona, Salerno, Italy; ^16^ AdRes Health Economics and Outcome Research, Turin, Italy; ^17^ Roche S.p.A., Monza, Italy; ^18^ Department of Public Health, University Federico II of Naples, Naples, Italy; ^19^ Department of Oncology, San Luigi Gonzaga Hospital, University of Turin, Orbassano, Italy; ^20^ Comprehensive Cancer Center, Fondazione Policlinico Universitario Agostino Gemelli Istituti di Ricovero e Cura a Carattere Scientifico (IRCCS), Roma, Italy; ^21^ Medical Oncology, Università Cattolica del Sacro Cuore, Roma, Italy

**Keywords:** next generation sequencing, non-small cell lung cancer, precision medicine, liquid biopsy, target therapy

## Abstract

**Introduction:**

To date, for all non-small cell lung cancer (NSCLC) cases, it is recommended to test for driver alterations to identify actionable therapeutic targets. In this light, comprehensive genomic profiling (CGP) with next generation sequencing (NGS) has progressively gained increasing importance in clinical practice. Here, with the aim of assessing the distribution and the real-world frequency of gene alterations and their correlation with patient characteristics, we present the outcomes obtained using FoundationOne (F1CDx) and FoundationLiquid CDx (F1L/F1LCDx) NGS-based profiling in a nationwide initiative for advanced NSCLC patients.

**Methods:**

F1CDx (324 genes) was used for tissue samples, and F1L (70 genes) or F1LCDx (324 genes) for liquid biopsy, aiming to explore the real-world occurrence of molecular alterations in aNSCLC and their relationship with patients’ characteristics.

**Results:**

Overall, 232 advanced NSCLC patients from 11 Institutions were gathered [median age 63 years; never/former or current smokers 29.3/65.9%; adenocarcinoma/squamous 79.3/12.5%; F1CDx/F1L+F1LCDx 59.5/40.5%]. Alterations were found in 170 different genes. Median number of mutated genes per sample was 4 (IQR 3–6) and 2 (IQR 1–3) in the F1CDx and F1L/F1LCDx cohorts, respectively. *TP53* (58%), *KRAS* (22%), *CDKN2A/B* (19%), and *STK11* (17%) alterations were the most frequently detected. Actionability rates (tier I and II) were comparable: 36.2% F1CDx vs. 34% ctDNA NGS assays (29.5% and 40.9% F1L and F1LCDx, respectively). Alterations in *KEAP1* were significantly associated with *STK11* and *KRAS*, so as *TP53* with *RB1*. Median tumor mutational burden was 6 (IQR 3–10) and was significantly higher in smokers. Median OS from metastatic diagnosis was 23 months (IQR 18.5–19.5) and significantly lower in patients harboring ≥3 gene mutations. Conditional three-year survival probabilities increased over time for patients profiled at initial diagnosis and exceeded those of individuals tested later in their clinical history after 12 months.

**Conclusion:**

This study confirms that NGS-based molecular profiling of aNSCLC on tissue or blood samples offers valuable predictive and prognostic insights.

## 1 Background

Lung cancer is the second most common cancer in the world and the first cause of cancer-related deaths (1). In Italy, lung cancer ranks second and third in incidence in men (15% of all tumors) and women (6% of all tumors), respectively, and accounted for 34,000 estimated deaths in 2021 (2). Histologically, most cases are categorized as non-small-cell lung cancer (NSCLC) and approximately half of the patients are diagnosed with metastatic disease (3).

The therapeutic landscape has evolved to encompass oncogene-addicted (15–20%), for whom molecularly targeted agents are available over multiple lines of treatment, and non-oncogene addicted disease (80–85%), for whom immune checkpoint blockade, alone or combined with chemotherapy, is the preferred treatment path (4), resulting in a dramatic change in survival and quality of life for advanced NSCLC patients.

Most actionable oncogenic alterations occur in lung adenocarcinoma (LUAD), the most common NSCLC subtype (5, 6). Actionable drivers in lung LUAD involve the epidermal growth factor receptor (*EGFR*), *KRAS*, *ALK* genes and, less commonly, *ROS1*, *BRAF*, *MET*, *RET*, *ERBB2*, *NTRK*, and *NRG1* (5, 6). Drivers found in squamous cell carcinoma (SCC, accounting for 35% of NSCLC patients) are much less frequently actionable and include *TP53*, *PIK3CA*, *CDKN2A*, *SOX2*, and *CCND1* alterations (4).

Testing for driver alterations is recommended for all NSCLC cases to find actionable therapeutic targets (6). To this purpose, different techniques may be used: immunohistochemistry (IHC), fluorescence *in situ* hybridization (FISH), multiplex reverse transcriptase-polymerase chain reaction (RT-PCR) panel assays, *in situ* hybridization (ISH), or comparative genomic hybridization. However, all relevant molecular alterations may also be detected by next generation sequencing (NGS). In that respect, ESMO guidelines (6) state that “if available, multiplex platforms (NGS) for molecular testing are preferable”, favoring the use of RNA- or DNA-based NGS designed to capture gene fusions (7).

Comprehensive genomic profiling (CGP) with NGS allows for multigene sequencing in a unique sample, identification of structural variants and genomic signatures and has predictive, prognostic, and therapeutic impact. Although the benefit of broad-based genomic sequencing, defined as any multigene panel testing more than 30 genes, remains debated (8), CGP is becoming increasingly easy and cheap, thus growing in utility with a larger number of patients (9). Previously unreported alterations are now routinely found, thus making it difficult to interpret genomic testing, as only 2% of somatic alterations are known oncogenic events (9). Moreover, non-invasive testing using blood as the source biological material and circulating free DNA (cfDNA) or circulating tumor DNA (ctDNA) as the substrate for CGP (liquid biopsy) is becoming increasingly used as an alternative to tissue-based profiling (10).

FoundationOne^®^CDx (F1CDx; Foundation Medicine, Inc., Cambridge, MA, USA) is a qualitative, NGS-based *in vitro* diagnostic test (11). Using a targeted high throughput hybridization-based capture technology, it is able to detect substitutions, insertion and deletion alterations (indels), copy number alterations (CNAs), and gene rearrangements in 324 genes, genomic biomarkers including microsatellite instability (MSI), and tumor mutational burden (TMB) (11). Source material is formalin-fixed, paraffin-embedded (FFPE) tumor tissue specimens (11) FoundationOne^®^Liquid (F1L; Foundation Medicine, Inc., Cambridge, MA, USA) and FoundationOne^®^Liquid CDx (F1LCDx; Foundation Medicine, Inc., Cambridge, MA, USA) are other NGS-based *in vitro* diagnostic tests (12) that use targeted high throughput hybridization-based capture technology to detect and report alterations in 70 and 324 genes, respectively, from plasma derived from anti-coagulated peripheral whole blood.

The IMpact of broad genoMic profIling oN advancEd NSCLC ouTcome (IMMINENT) study aimed at assessing the distribution and the real-world frequency of gene alterations and their correlation with patient characteristics in a population of advanced NSCLC patients; it also sought to evaluate the clinical impact of molecular profiling and the timing of CGP with respect to the line of treatment.

## 2 Materials and methods

### 2.1 Study design and informed consent statement

This retrospective real-world data analysis leveraged a clinical-genomic database including anonymized patient-level data of NSCLC patients who underwent CGP within a national FoundationOne access program in 11 Italian Oncological centers between May 2019 and November 2022. Data had been previously collected and stored by the University Hospital Trust in Verona in accordance with the Helsinki declaration, as all the patients signed the informed consent form (ICF) for secondary use of their data for research purposes. Data included clinico-pathological features, treatment history, and tumor profiling results. Genomic analysis was performed using the F1CDx assay on tumor tissue specimens or using circulating tumor DNA (ctDNA) profiling assays, including F1L and F1LCDx (the latter launched in August 2020) on DNA from blood sampling.

### 2.2 Inclusion and exclusion criteria

Patients included in this study were adults with histologically confirmed advanced NSCLC diagnosis who were profiled by NGS test using tissue biopsies (F1CDx) or blood samples (F1L and F1LCDx).

Exclusion criteria were patients<18 years of age, lack of medical reports, and unwillingness or impossibility to sign the written ICF.

### 2.3 NGS analysis

F1CDx analyzes DNA extracted from formalin-fixed paraffin-embedded (FFPE) tumor samples. Specimens with at least 50 ng of DNA were used for library construction. Hybridization capture and multiplex sequencing were performed to a mean coverage depth of >550X for 324 cancer-related genes. Sequencing data were processed using a proprietary bioinformatics pipeline, which was designed to detect base substitutions, indels, CNAs, gene rearrangements, TMB, and MSI (13–15).

For F1L and its new version F1LCDx assays, circulating free DNA (cfDNA) was obtained from plasma derived from peripheral whole blood. Extracted cfDNA underwent whole-genome shotgun library construction and hybridization-based capture of 70 and 324 genes. Then, selected libraries were sequenced with deep coverage (median depth >6000x).

Testing were conducted to evaluate the impact of a range of cfDNA input masses (50% below the lower limit and 33% above the upper limit) for F1LCDx using an updated library construction input range (20-60ng).

The assays reported base substitutions, indels, and selected CNAs and gene rearrangements (16, 17). F1LCDx also detected genomic signatures, such as MSI and blood TMB (bTMB), while F1L reported only MSI.

Only known or likely pathogenic alterations were considered in this study.

TMB was measured by counting somatic, non-driver coding mutations per megabase (mut/Mb) of coding genome (14, 17). The MSI status was based on genome wide analysis across >2000 microsatellite loci, and was generated by calculating the fraction of unstable ones (containing a repeated length not present in an internal database generated using >3000 clinical samples) (14, 17).

### 2.4 Actionability classification

Genomic alterations classified as tier I-III mutations according to ESCAT (6, 7) were analyzed.

### 2.5 Statistical analysis

Statistical analysis was performed using R (version 4.1.2). Descriptive statistics included percentages and frequencies for categorical variables, and median and interquartile range (IQR) for continuous variables. Categorical variables were compared using the χ^2^ or Fisher exact test when appropriate, while continuous variables were compared using the Mann-Whitney U or Kruskal Wallis test. Comparisons between proportions were analyzed by Fisher’s exact test. Multiple testing correction was applied using the Benjamin-Hochberg (BH) method. The overall survival from histological (hOS) and metastatic (mOS) diagnosis were analyzed using Kaplan-Meier (KM) method, and the log rank test was used to compare the survival curves among the groups. Conditional survival (CS) was computed using the multiplicative law of probability. *CS*(*y*|*x*) can be defined as the probability of surviving additional *y* years, given that the patient has already survived *x* years. It can be expressed as:


$$CS\left(y|x\right)=\frac{S\left(x+y\right)}{S\left(x\right)}$$

A p-value<0.05 was considered statistically significant.

## 3 Results

### 3.1 Patient characteristics

A total of 246 NSCLC patients were profiled by tissue- and ctDNA-based NGS assays (F1CDx, F1L, and F1LCDx). Tissue-based F1CDx assay was performed in 146 (59.3%) patients, while the remaining 100 (40.7%) were liquid biopsy samples, 78 (78%) and 22 (22%) of which tested by F1L and F1LCDx, respectively. The proportion of testing failure (no results) was 5.5% (8 out of 146) for tissue sequencing and 6% (6 out of 100) for ctDNA testing (all with F1L). Overall, analyses were completed for 232 samples, with a success rate of 94.3%. For those patients in whom molecular profiling failed, further analysis through liquid biopsy was not proposed, as per protocol.

**Table 1** and **Supplementary Data 1**, **Supplementary Figure S1**, summarize patients’ clinico-pathological characteristics; differences between the patients tested with tissue- and ctDNA-based NGS were statistically significant.

**Table 1:** Clinical characteristics of NSCLC patients.

Variable	Total (N=232)	F1CDx (N=138, 59.5%)	F1L/F1LCDx (N=94, 40.5%)
Sex (male) - N (%)	122 (52.6%)	87 (63%)	35 (37.2%)
Age - Median (IQR)*	63 (55.3–69.8)	64 (56.8–70)	61 (53.3–66.8)
Smoking status - N (%)			
Never-smoker	68 (29.3%)	25 (18.1%)	43 (45.7%)
Ever-smoker	153 (65.9%)	103 (74.6%)	50 (53.2%)
NA	11 (4.7%)	10 (7.3%)	1 (1.1%)
Pack/year smoking history - Median (IQR)^	10 (0–35)	30 (6.8–47.8)	3 (0–9)
ECOG performance status - N (%)			
0	130 (56%)	68 (49.3%)	62 (66%)
1	85 (36.6%)	55 (39.9%)	30 (31.9%)
2	16 (6.9%)	14 (10.1%)	2 (2.1%)
3	1 (0.4%)	1 (0.7%)	–
Histology - N (%)			
LUAD	184 (79.3%)	100 (72.5%)	84 (89.4%)
SCC	29 (12.5%)	22 (15.9%)	7 (7.5%)
Other	16 (6.9%)	13 (9.4%)	3 (3.2%)
NA	3 (1.3%)	3 (2.2%)	
Metastases at histological diagnosis - N (%)^§^	154 (70%)	81 (60.4%)	73 (84.9%)
Number of metastatic sites - Median (IQR)	1 (0–2)	1 (0–2)	2 (1–3)
Stage at histological diagnosis - N (%)			
I-II	18 (7.8%)	14 (10.2%)	4 (4.3%)
III	49 (21.1%)	38 (27.5%)	11 (11.7%)
IV	161 (69.4%)	83 (60.2%)	78 (83%)
NA	4 (1.7%)	3 (2.2%)	1 (1.1%)

*2 missing values; ^27 missing values; ^§^12 missing values. LUAD, adenocarcinoma; ECOG, Eastern Cooperative Oncology Group; IQR, interquartile range; N, number; NA, not available; SCC, squamous cell carcinoma.

### 3.2 Mutational profile of NSCLC

**Figure 1:**
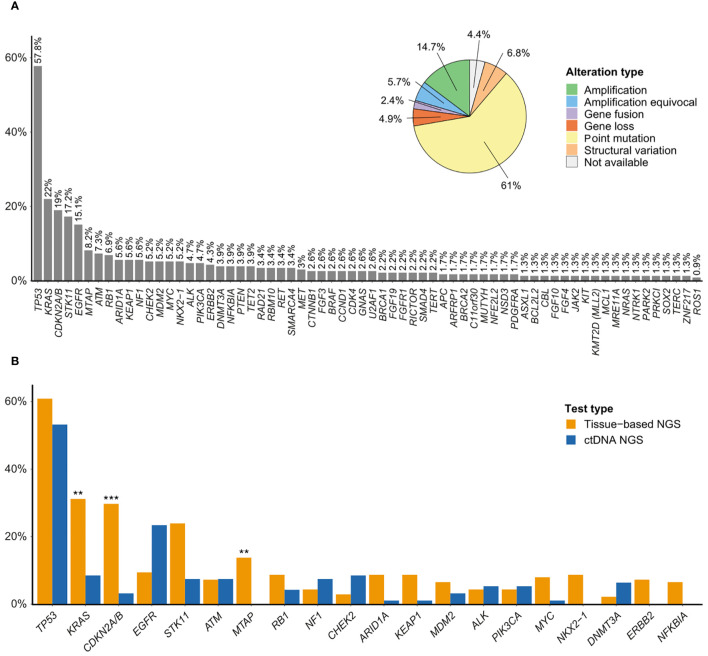
**(A)** Mutation profile of NSCLC. Figure shows driver genes and genes mutated in at least 1% of the patients, and distribution of alteration types. **(B)** Comparison of gene frequencies between tissue-based NGS (n=138) and ctDNA NGS (n=94) (top-20 mutated genes). The Y-axis indicates the percentage of patients with the mutated genes across the X-axis for groups under study. **Adjusted p-value<0.01; ***Adjusted p-value<0.001.

A total of 923 genomic alterations were detected in 170 genes, with a median number of altered genes per sample of 3 (IQR: 2–5). Of the 232 successfully tested samples, 219 (94.4%) exhibited one or more genomic alterations, with a significantly lower number of altered genes per sample detected by F1L, as compared to F1CDx and F1LCDx (p<0.001, **Supplementary Data 1**, **Supplementary Figure S2**). The genomic profile of the analyzed NSCLC samples is presented in **Figure 1A**.

The top frequently altered genes included *TP53* (57.8%), *KRAS* (22%), *CDKN2A/B* (19%; comprising 50% gene loss and 9.1% structural variation), *STK11* (17.2%), and *EGFR* (15.1%). Most variant types were point mutations (61%), followed by amplifications (14.7%), and structural variations (6.8%), which included insertions, deletions, truncations, large-scale gene losses, and gene rearrangements.

The comparison of gene frequencies between solid and liquid biopsy is presented in **Figure 1B** and **Supplementary Data 1**, **Supplementary Table S1**. Notable differences between tissue-based and ctDNA NGS included *KRAS* (31.2% vs. 8.5%, p<0.01), *CDKN2A/B* (29.7% vs. 3.2%, p<0.01), and *MTAP* (13.8% vs. 0%, p<0.01). Furthermore, ctDNA sequencing did not detect any alterations in *NKX2-1*, *ERBB2*, and *NFKBIA*. Mutations in *EGFR* were detected more frequently in liquid biopsy samples compared to tissue specimens, albeit the differences were non-significant, after adjusting ​​for multiple comparisons.

For the top-20 mutated genes, associations between mutations and clinical characteristics are depicted in **Supplementary Figures S3A–C**. *KRAS* and *STK11* mutation rates were more frequent in ever- than in never-smokers (*KRAS*: 29.4% ever-smoker vs. 7.4% never-smoker, p=0.03; *STK11*: 22.2% vs. 4.4%, p=0.06). Conversely, *ALK* alterations were more frequent in never-smokers, although this difference did not reach statistical significance (2.3% in ever-smokers vs. 11.8% in never-smokers, p=0.09). Additional information on the comparison of gene alteration frequencies is presented in **Supplementary Data 1**, **Supplementary Tables S2**–**S4**.

### 3.3 Actionability

**Figure 2:**
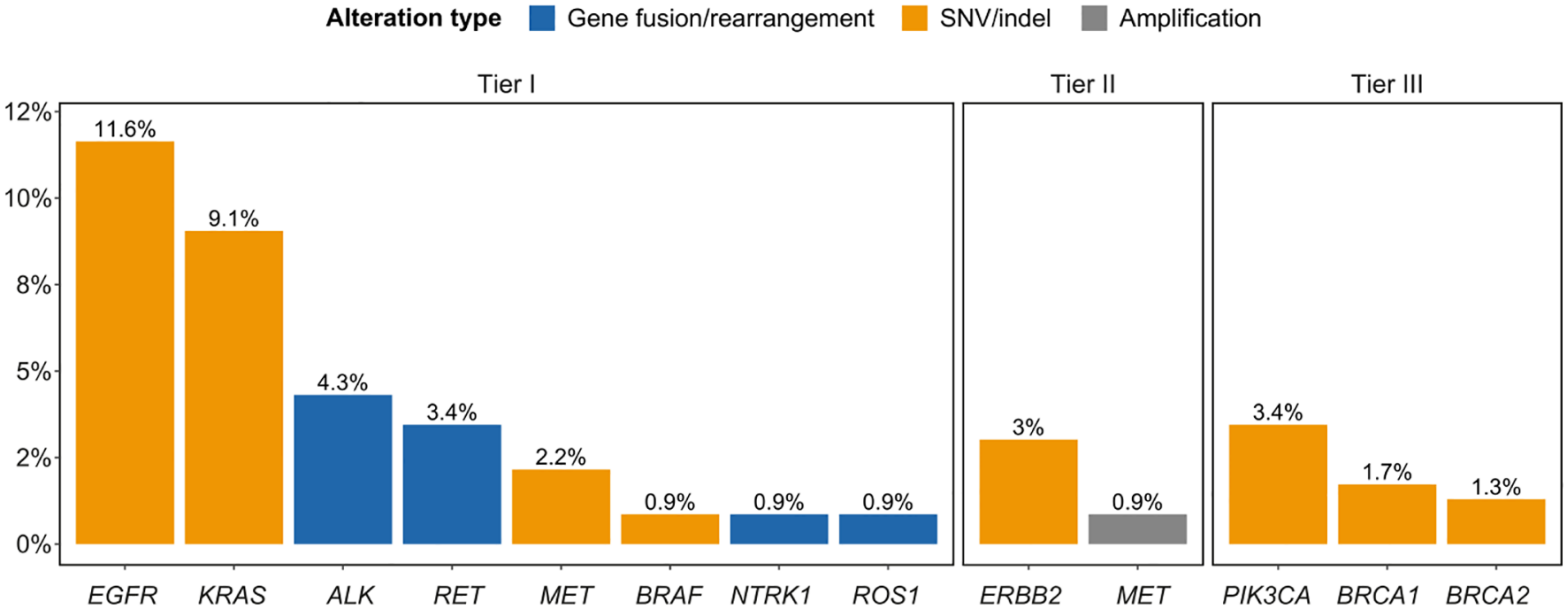
Frequencies of ESCAT tier I-III alterations. SNV, single nucleotide variant.

Frequencies and types of oncogenic driver alterations across all samples are presented in **Supplementary Data 1**, **Supplementary Table S1** and **S5**. Based on the ESCAT actionability scale, we identified 82 cases (35.3%) harboring at least one actionable gene alteration (74 [31.9%] tier I and 8 [3.5%] tier II), and 10 (4.3%) additional cases had an ESCAT tier III biomarker (**Figure 2**). Identified targetable genomic variants were: EGFR mutations (3.4%) and structural variations (including exon 19 deletion and exon 20 insertion, 8.6%; considering that in some patients both were detected, the total patients involved by EGFR mutations were 11.6%), KRAS mutations (G12C: 9.1%), ALK fusions (3.9%) and rearrangements (0.4%), RET fusions (3.4%, with 0.4% of patients carrying also a rearrangement in the same gene), MET exon 14 skipping mutations (2.2%) and amplifications (0.9%), BRAF mutations (V600E: 0.9%), NTRK1 fusions (0.9%, with 0.4% of patients carrying also a rearrangement in the same gene), ROS1 fusions (0.9%), and ERBB2 mutations (3%). The frequency of ESCAT tier III mutations was 3.4% for PIK3CA, 1.7% for BRCA1, and 1.3% for BRCA2.

Actionability rates (tier I and II) were comparable across panel types: 36.2% F1CDx vs. 34% ctDNA NGS assays (29.5% and 40.9% F1L and F1LCDx, respectively).

### 3.4 TMB and MSI status

**Figure 3:**
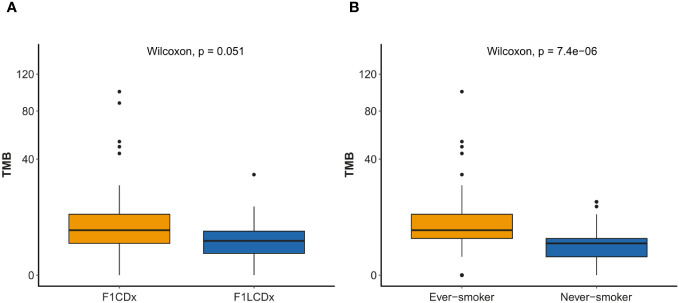
TMB comparison of genomic testing and smoking status. Y-axis presented in square root transformation. **(A)** F1CDx (n=123) vs. F1LCDx (n=22). **(B)** Ever-smoker (n=101) vs. never-smoker (n= 37). TMB, tumor mutational burden.

TMB was evaluable for 123 (89.1%) and 22 (100%) patients profiled by F1CDx and F1LCDx respectively. The difference in TMB between the two groups was on the verge of statistical significance, with a higher TMB in tissue specimens compared to liquid biopsy samples (median TMB: 6 for F1CDx vs. 3.5 for F1LCDx, p=0.051) (**Figure 3A**). In the whole cohort, the median TMB was 6 mut/Mb (IQR: 3–10) and 28.3% of patients exhibited TMB ≥10 mut/Mb. Significantly higher TMB was observed in ever-smokers (**Figure 3B**); TMB also tended to be higher in SCC tumors (vs. LUAD; **Supplementary Figure S4**).

Except for 2 cases with microsatellite instability, all tumors for which MSI status was available (135, 58.2%) were stable. The proportion of undetermined MSI status was significantly higher in liquid biopsy samples compared to tissue specimens (**Supplementary Data 1**, **Supplementary Figure S5**, p<0.001), as, by design, specimens assayed using F1L are reported as “Unknown” if MSI-H (high) is not detected.

### 3.5 Co-occurrence

**Figure 4:**
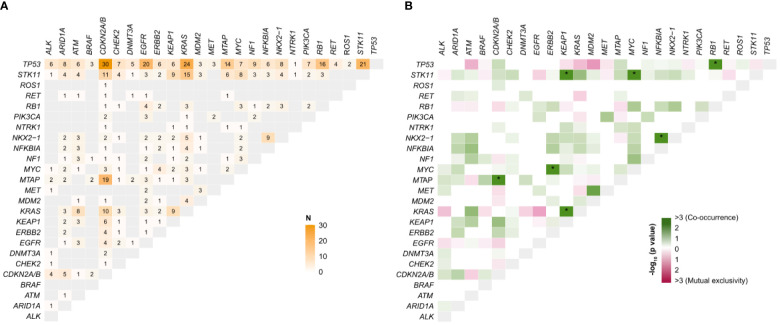
**(A)** Number of co-occurrences between gene pairs. **(B)** Mutual exclusivity and co-occurrence of top-20 genes/driver genes in 232 NSCLC tumors. P-values (not adjusted) were calculated using Fisher’s exact test and transformed into a score, -log10(P-value). *Adjusted p-value<0.05; N, number.

We investigated the distribution of co-occurring alterations in driver genes (**Supplementary Data 1**, **Supplementary Figure S6**) and analyzed the statistical significance of mutual exclusivity and co-occurrence of the most frequent variants in this cohort (**Figure 4**).

Concurrently mutated genes were identified in 187 (80.6%) patients. *TP53* represented the most prevalent co-alteration in all tumors (**Figure 4A**) and significantly co-occurred with *RB1* (**Figure 4B**, p<0.01). *KEAP1* co-mutations were highly represented in tumors with *KRAS* (9/51, 17.6%; p=0.01) and *STK11* (9/40, 22.5%; p<0.01) alterations. Despite the propensity for mutual exclusivity, we discovered 3 (5.9%) and 24 (47.1%) *KRAS*-mutant patients harboring *EGFR* and *TP53* alterations, respectively. The spectrum of *KRAS* co-mutations also comprised aberrations in *STK11* (15/51, 29.4%) and *CDKN2A/B* (10/51, 19.6%) (**Supplementary Data 1**, **Supplementary Figure S6A**). Among *EGFR*-mutant patients*, TP53* alterations were detected in 57.1% (20/35) of cases, followed by *PTEN* (5/35, 14.3%), *CDKN2A/B* (4/35, 11.4%), and *RB1* (4/35, 11.4%) (**Supplementary Data 1**, **Supplementary Figure S6B**). *ERBB2*-mutant patients were significantly enriched with *MYC* (4/10, 40%; p=0.03), whereas *ALK* mostly co-occurred with *TP53* (6/11, 54.5%) and *CDKN2A/B* (4/11, 36.7%) (**Supplementary Data 1**, **Supplementary Figures S6C, D**). *MTAP*/*CDKN2A/B*, *MYC*/*STK11*, and *NFKBIA*/*NKX2-1* were other co-occurrent variants with statistical significance. These results mostly apply to patients who underwent tissue biopsy. Due to the low number of cases, patients profiled by F1L/F1LCDx exhibited a less obvious pattern (**Supplementary Data 1**, **Supplementary Figure S7**), with no significant co-occurrences and a non-significant tendency of *MET*-mutated tumors towards *MDM2* enrichment.

### 3.6 Overall survival

**Figure 5:**
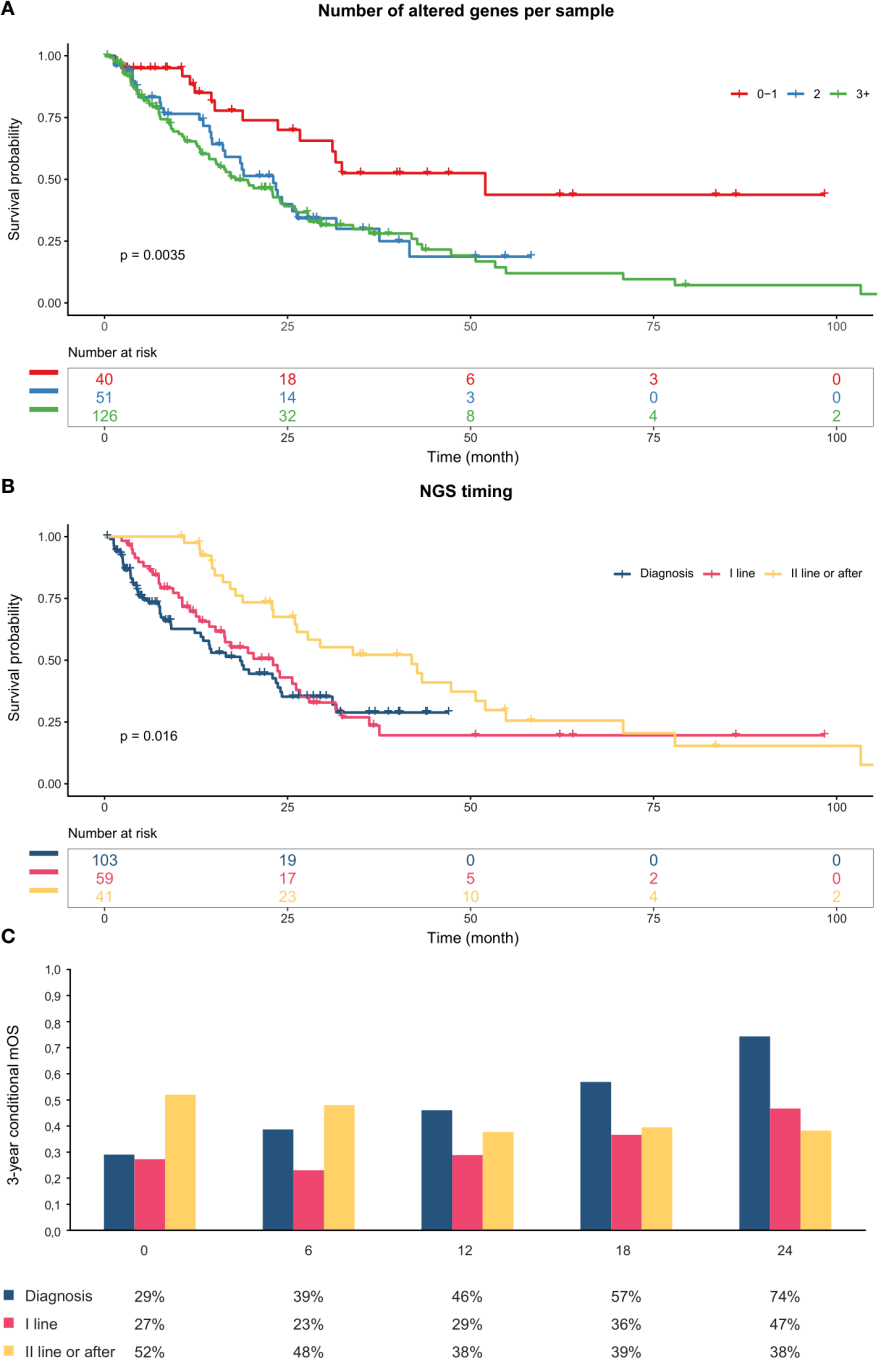
**(A)** Kaplan-Meier Curves indicating overall survival from metastatic diagnosis stratified by number of altered genes per sample. **(B)** Kaplan-Meier Curves indicating overall survival from metastatic diagnosis stratified by NSG timing. **(C)** Three-year conditional mOS by NGS timing. The x-axis represents the duration of survival to date (6-month intervals). All patients in the “Diagnosis” group died within 42 months; therefore, they were assumed to have a constant survival rate from that point onwards. mOS, overall survival from metastatic diagnosis.

Survival data were available for 229 (98.7%) patients, 217 of whom (94.6%) with metastatic disease. The median hOS and mOS were 27.8 (95% CI: 24.2–42.7) and 23 (95% CI: 18.5–26.7) months, respectively, with a 5-year survival rate of 29.3% (hOS; 95% CI: 22.1–39%) and 18.6% (mOS; 95% CI: 12.1–28.7%). In an explorative univariate analysis, a higher number of altered genes was associated with worse hOS and mOS (p<0.01; **Figure 5**, **Supplementary Figure S8G**).

Never-smokers had longer hOS than those with a smoking history (p = 0.041; **Supplementary Data 1**, **Supplementary Figure S8E**), and patients with stage at diagnosis I-II had longer hOS than patients who were diagnosed with stage III or IV (p<0.0001; **Supplementary Data 1**, **Supplementary Figure S8A**). Kaplan-Meier curves also indicated poorer survival in patients with higher ECOG PS (p<0.0001; **Supplementary Data 1**, **Supplementary Figures S8C, D**).

We also analyzed the impact of the timing of NGS tests on OS. The majority of the patients underwent single-gene analysis, at least for EGFR evaluation (94% of patients), at the time of diagnosis. Most NGS tests (114, 49.1%) were performed after initial diagnosis or before starting the first line of therapy; 59 (25.4%) were performed when first-line treatment was ongoing; all others were performed before or during subsequent lines (41, 17.7%). For 18 (7.8%), this information was not available.

Patients who were profiled at or after second line display longer mOS, as compared with subjects tested at the time of diagnosis or during the first-line treatment (**Figure 5B**). However, conditional one- and three-year survival probabilities increased over time for patients profiled at initial diagnosis and exceeded those of individuals tested later in their clinical history after 36 and 12 months, respectively (**Figure 5C**, **Supplementary Data 1**, **Supplementary Figure S9**).

## 4 Discussion

In this study, we analyzed the results of CGP performed on either tissue or liquid biopsy samples in a real-world population of Italian NSCLC patients. The patient population analyzed was strikingly similar to the NSCLC population recently reported in the context of the RATIONAL Italian registry study (18), encompassing advanced disease, 85% adenocarcinoma histology, and approximately 45% of tests performed at diagnosis or at I-line treatment start. These figures reflect current indications for NGS testing in Italy (5). In line with indications to liquid biopsy at the time of testing, the population of patients who underwent ctDNA NGS was significantly skewed towards younger, female, adenocarcinoma patients, less heavily exposed to cigarette smoke, who were metastatic at diagnosis. Regardless of the sample source (tissue or ctDNA), NGS tools had a very high success rate in sequencing, i.e., a mean of 94.3%, which is in line with the relevant technical information and other reported series (11, 12, 19, 20).

The number and types of gene alterations (in particular those falling in ESCAT tiers I/II) reported in our experience are in line with the literature (6, 7). *MET* alterations were slightly less prevalent in our cohort, primarily due to the lower occurrence of high-level amplifications (0.9% compared with 2-4% reported in the literature (21, 22)). This discrepancy may be due to the rarity of these mutations and the low sample size of our cohort, as well as to the lower ability of ct-DNA-based methods (employed in approximately 41% of our cohort) to detect *MET* amplifications (23). Exon 14 skipping mutation rate (2.2%) was consistent with that reported in the literature (3%) (6).

Significant differences between tissue-based and ctDNA NGS included *KRAS*, *CDKN2A/B*, and *MTAP*. *KRAS* frequency was significantly higher in cases tested with the tissue-based F1CDx than in those tested by ctDNA-based panels (F1L/F1LCDx). This may be due to a selection bias skewing the population subjected to liquid testing towards second or further lines and enriching it for *EGFR*-mutant cases progressing on TKI treatment; indeed, the prevalence of smokers was significantly lower (74.6% vs 53.2%) and that of EGFR mutations was higher (9.4% vs 23.4%) in patients tested by liquid biopsy. Genes like *CDKN2A/B* and *MTAP* may have an artificially low prevalence in this type of study, due to copy number deletions not being detected by liquid assays. Similarly, no alterations in *NKX2-1*, *ERBB2*, and *NFKBIA* were found using ctDNA sequencing, possibly due to lower sensitivity of liquid biopsy to detect *ERBB2* copy number alterations (23) and the fact that *NKX2-1* and *NFKBIA* are not baited on F1L test. Part of the differences in detection rates observed with liquid, as opposed to tissue-based tests, may also be attributable to the size of the panel used; indeed, even though the sample size in the F1LCDx was low, we observed a higher proportion of actionable genes detected by this test compared to the older F1L (40.9% vs. 29.5%), presumably due to the greater number of genes analyzed (324 vs. 70). It seems that the larger the gene panel, the higher the proportion of actionable genes detected (24, 25). The increase in the percentage of actionable genes ranged from 31% (24) to 45% (25). In addition, F1LCDx was shown to have a higher success rate than F1L (26).

In addition to IHC-based PD-L1 expression, high TMB, generally defined as TMB ≥ 10 mutations/Mb, may predict response to immunotherapy (7, 27), although this is not yet a recognized regulatory biomarker in Europe (7). In our cohort, 28% of 145 TMB-assessable patients had high TMB (30.1% in tissue biopsies); such frequency is slightly lower than that reported in the literature for NSCLC (36%), possibly due to differences between tests. Indeed, greater TMB was detected by F1CDx than by F1LCDx; such difference may be explained by a relatively lower prevalence of smokers in the liquid cohort (a greater frequency of high TMB is observed among ever-smokers in our cohort, consistent with other reports (28)) and/or by the possibility that a low tumor fraction may lead to TMB underestimation in blood samples (29). MSI status is another predictive biomarker for immunotherapy (30). In our study, a greater proportion of patients (86%) had undetermined MSI status in liquid specimens when compared to tissue ones (12%). This is due to the design of F1L assay, which reports only MSI-H status. Genomic space covered and panel composition may result in a different efficiency in detecting MSI. However, in NSCLC MSI is rare (1%) and only a few studies have evaluated its impact on response to immunotherapy (31).

Another potential advantage of NGS is the ability to detect co-occurrences that may affect the response to anticancer therapies in NSCLC (32). *TP53* is one of the most important tumor suppressors (33), frequently mutated in almost all types of cancers, including NSCLC (34). Thus, it is not surprising that concomitant alterations of *TP53* and actionable genes may affect responsiveness to TKI and immunotherapy. In the IMMINENT cohort, *TP53* was the most prevalent co-alteration in all tumors, particularly in *EGFR*- and *KRAS*-mutated tumors. Co-occurrence of *TP53* and *EGFR* mutations is generally associated with worse prognosis in patients treated with TKIs (35, 36). In the IMMINENT cohort, co-occurrences were also significant with *RB1*; patients with *EGFR/RB1/TP53* co-mutant lung cancer are at risk of small or large cell neuroendocrine lung cancer transformation (37, 38). Although usually mutually exclusive, co-occurrence of *EGFR* and *KRAS* alterations (found in three patients in the IMMINENT cohort) can be found with highly sensitive methods and usually portends primary and/or acquired resistance to EGFR TKIs (39). In the IMMINENT dataset, *STK11* was the second most frequently co-mutated gene, among *KRAS*-mutant patients, although this association did not reach statistical significance. *STK11/KEAP1* and *KRAS/KEAP1* co-mutations have been reported by other studies (40), with variable association with clinical outcome (41, 42). To our knowledge, this is the first study reporting co-occurrences of *MYC/STK11* and *MYC/ERBB2* and were preliminarily confirmed as statistically significant in the TCGA pan-lung cancer cohort (43). Overall, the concept that co-mutations are associated with worse outcome is supported by an exploratory univariate analysis showing, in the IMMINENT cohort, a significantly better survival for patients whose tumors harbored no or a single genomic driver, as compared to those characterized by a more complex genomic landscape. These findings, however, need further analysis to exclude potential confounders: smoking history may play a role in the association between the number of altered genes and worse OS, as smokers display both a higher number of mutations and worse prognosis. Moreover, the association between a higher number of altered genes and worse hOS and mOS was not observed when the analysis was restricted to tissue specimens; in blood samples, the absence of mutations may indicate minimal or no shedding of tumor DNA, which may have positive prognostic value (44).

Survival outcomes for the IMMINENT population compare favorably with those reported in a recent Italian real-world experience (45). Better figures in the IMMINENT cohort, as compared to registry data, probably reflect a relative selection of patients, as well as the inclusion of patients who had been treated with first-line chemo-immunotherapy combinations (reimbursed in Italy since 2019). The clear impact of well-known prognostic factors (such as stage at diagnosis for hOS, ECOG PS, and smoking status for both h and mOS) attests to the representativeness of the population studied and to the generalizability of the results.

Current clinical guidelines advocate for the molecular genotyping of patients newly diagnosed with metastatic NSCLC. Real-world evidence underscores the significant survival benefits associated with comprehensive molecular profiling performed either at the time of diagnosis or before initiating first-line treatment. This approach ensures that patients receive tailored therapies targeting specific genetic alterations, thereby optimizing treatment efficacy and patient outcomes (46, 47). In our study, somewhat contrary to expectations, both h and mOS appeared to be significantly longer for patients tested in second or further lines; such finding may be explained in part by the fact that the probability of surviving for a defined further period of time is higher for patients who have already survived up to a certain landmark (conditional survival (48)); indeed, in our series conditional 3-year survival probability did not differ for patients tested with NGS at different times in their disease course, provided that they had already survived 1 year.

Additionally, single gene testing was conducted for the majority of the analyzed patients, allowing for targeted therapy initiation at diagnosis in many cases. However, previous single gene testing may have, in some instances, compromised the amount of available material for comprehensive genomic profiling (CGP) analysis (49).

This study has some limitations, which include its retrospective nature and a relatively small sample size. Another significant limitation is the unavailability of tumor fraction (TF) data in the liquid biopsy cohort. Specifically, for patients who underwent the F1L test, the TF data is not specified in the report. On the other hand, for patients who were subjected to the F1LCdx test, TF data was available for only 13 patients, out of which only 6 patients had high TF levels. This lack of comprehensive TF data is relevant given the clinical implications of TF levels. Patients with who have negative liquid biopsy results and a ctDNA TF of 1% or higher are unlikely to have a driver mutation detected on subsequent tissue testing. Therefore, these patients might benefit from starting treatment immediately. On the other hand, those with a negative liquid biopsy and a ctDNA TF of less than 1% often have a driver mutation identified in follow-up tissue testing and should be prioritized for additional analysis (50). Our limited availability of TF data constrains the ability to draw comprehensive conclusions about the correlation between TF levels and clinical outcomes in the study population. Of note, high-risk clonal hematopoiesis (CH) is often unexpectedly detected in solid tumor patients undergoing plasma cell-free DNA sequencing. These findings could lead to further hematologic diagnostic tests and uncover an occult hematologic malignancy (51). These insights pose a limitation to our study as we did not evaluate monoclonal components due to data insufficiency and the primary objectives of the study.

In that respect, other data sources such as the ongoing ATLAS registry [https://biomarkersatlas.com (52)], prospectively collecting data on the molecular characterization of NSCLC in the majority of Italian Centers, may overcome many of such limitations. Moreover, currently ongoing prospective trials, such as the Liquid-First trial (NCT05846594), will contribute to define the place of ctDNA-based NGS tests in the upfront molecular characterization of newly diagnosed, advanced NSCLC.

## 5 Conclusions

In conclusion, the IMMINENT study, analyzing data coming from both liquid and tissue NGS tests performed on 246 patients affected by NSCLC in the period May 2019–November 2022, confirms the utility of CGP in the upfront molecular characterization of advanced NSCLC. Further analysis will help gaining insights into prognostic and predictive value of specific genomic alterations or combinations thereof.

## Data availability statement

The datasets presented in this study can be found in online repositories. The names of the repository/repositories and accession number(s) can be found in the article/**Supplementary Material**.

## Ethics statement

The study was conducted in accordance with the Declaration of Helsinki, and approved by the Institutional Ethics Committee of clinical research of AOUI Verona (protocol code 59115 of 11/10/2021). The studies were conducted in accordance with the local legislation and institutional requirements. The participants provided their written informed consent to participate in this study.

## Author contributions

MaS: Conceptualization, Data curation, Investigation, Methodology, Resources, Validation, Writing – original draft, Writing – review & editing. LB: Conceptualization, Data curation, Investigation, Methodology, Resources, Validation, Writing – original draft, Writing – review & editing. RN: Conceptualization, Data curation, Investigation, Methodology, Resources, Validation, Writing – original draft, Writing – review & editing. JI: Data curation, Writing – review & editing. IS: Data curation, Writing – review & editing. JM: Data curation, Writing – review & editing. MiS: Data curation, Writing – review & editing. AL: Data curation, Writing – review & editing. FB: Data curation, Writing – review & editing. FV: Data curation, Writing – review & editing. FS: Data curation, Writing – review & editing. GA: Data curation, Writing – review & editing. LC: Data curation, Writing – review & editing. MO: Data curation, Writing – review & editing. DM: Data curation, Writing – review & editing. AV: Data curation, Writing – review & editing. FL: Data curation, Writing – review & editing. HP: Data curation, Writing – review & editing. FF: Data curation, Writing – review & editing. CS: Data curation, Writing – review & editing. CP: Data curation, Formal analysis, Methodology, Writing – original draft, Writing – review & editing. LP: Data curation, Formal analysis, Methodology, Writing – original draft, Writing – review & editing. ES: Conceptualization, Data curation, Methodology, Writing – review & editing. SC: Conceptualization, Data curation, Methodology, Writing – review & editing. UM: Supervision, Validation, Visualization, Writing – review & editing. SN: Supervision, Validation, Visualization, Writing – review & editing. EB: Supervision, Validation, Visualization, Writing – review & editing. SP: Conceptualization, Data curation, Methodology, Supervision, Validation, Visualization, Writing – original draft, Writing – review & editing. MM: Conceptualization, Data curation, Supervision, Validation, Visualization, Writing – original draft, Writing – review & editing.

## Glossary

cfDNA: circulating free DNA
CGP: comprehensive genomic profiling
CNAs: copy number alterations
CS: conditional survival
ctDNA: circulating tumor DNA
EGFR: epidermal growth factor receptor
ESMO: European Society for Medical Oncology
FFPE: formalin-fixed paraffin-embedded
F1CDx: FoundationOne^®^CDx
F1L: FoundationOne^®^Liquid
F1LCDx: FoundationOne^®^Liquid CDx
FISH: fluorescence *in situ* hybridization
GCN: gene copy number
hOS: overall survival from histological diagnosis
ICF: informed consent form
IHC: immunohistochemistry
IMMINENT: IMpact of broad genoMic profIling oN advancEd NSCLC outcome
IQR: interquartile range
ISH: *in situ* hybridization
KM: Kaplan-Meier
LUAD: adenocarcinoma
mOS: overall survival from metastatic diagnosis
MSI: microsatellite instability
MS: microsatellite status
NGS: next generation sequencing
NSCLC: non-small-cell lung cancer
PD-L1: programmed death-ligand 1
RT-PCR: reverse transcriptase-polymerase chain reaction
SCC: squamous cell carcinoma
SCLC: small-cell lung cancer
SNV: single nucleotide variations
TCGA: The Cancer Genome Atlas
TMB: tumor mutational burden
