# Supplementary material for: Tissue- and liquid-biopsy based NGS profiling in advanced non-small-cell lung cancer in a real-world setting: the IMMINENT study
Source: Front Oncol. 2024 Jul 9;14:1436588. doi: 10.3389/fonc.2024.1436588 (PMC11263796; doi:10.3389/fonc.2024.1436588)

Figure S1. (**A**) Distribution of other histological subtypes (16 cancer patients). (**B**) Metastatic sites.

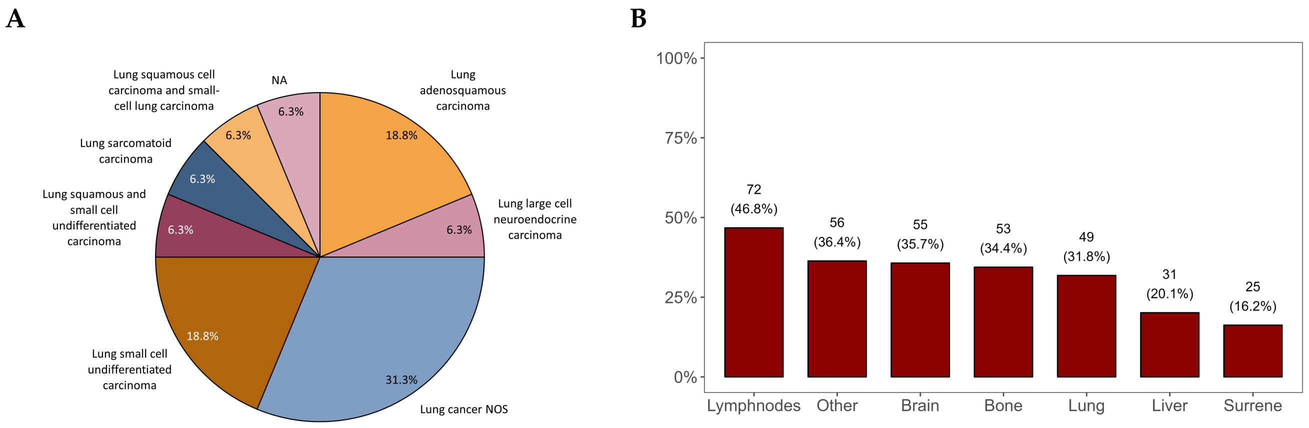

Figure S2. Number of altered genes per sample. (**A**) Distribution in the entire cohort. (**B**) Tissue-based NGS (n=138) vs. ctDNA NGS (n=94). N: number.

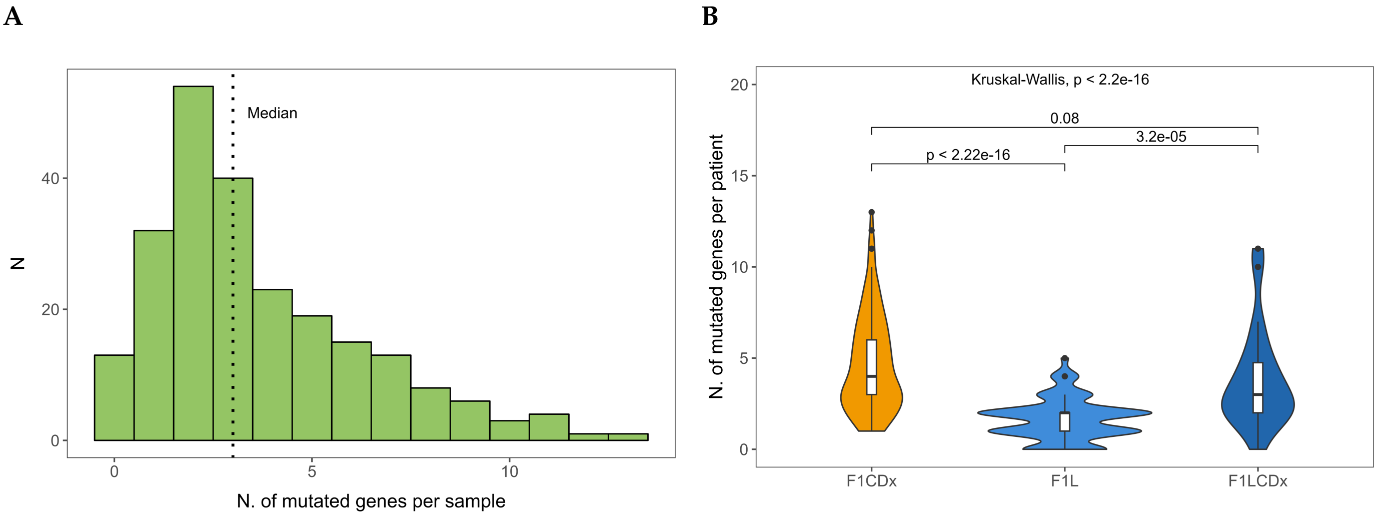

Table S1. Comparison of gene frequencies between F1LCDx and F1L/F1LCDx. Selected genes altered in at least 1% of the cohort. Driver genes in NSCLC with frequency <1% were also included.

Gene	F1CDx (N=138)	F1L/F1LCDx (N=94)	P-value	Adjusted p-value
TP53	84 (60.9%)	50 (53.2%)	0.279	1
KRAS	43 (31.2%)	8 (8.5%)	0	0.002
CDKN2A/B	41 (29.7%)	3 (3.2%)	0	0
STK11	33 (23.9%)	7 (7.4%)	0.001	0.052
EGFR	13 (9.4%)	22 (23.4%)	0.005	0.135
MTAP	19 (13.8%)	0 (0%)	0	0.002
ATM	10 (7.2%)	7 (7.4%)	1	1
RB1	12 (8.7%)	4 (4.3%)	0.291	1
KEAP1	12 (8.7%)	1 (1.1%)	0.017	0.288
NF1	6 (4.3%)	7 (7.4%)	0.387	1
ARID1A	12 (8.7%)	1 (1.1%)	0.017	0.288
NKX2-1	12 (8.7%)	0 (0%)	0.002	0.063

MDM2	9 (6.5%)	3 (3.2%)	0.369	1
CHEK2	4 (2.9%)	8 (8.5%)	0.073	0.647
MYC	11 (8%)	1 (1.1%)	0.03	0.43
PIK3CA	6 (4.3%)	5 (5.3%)	0.761	1
ALK	6 (4.3%)	5 (5.3%)	0.761	1
ERBB2	10 (7.2%)	0 (0%)	0.006	0.154
DNMT3A	3 (2.2%)	6 (6.4%)	0.164	0.959
TET2	4 (2.9%)	5 (5.3%)	0.491	1
NFKBIA	9 (6.5%)	0 (0%)	0.012	0.254
PTEN	6 (4.3%)	3 (3.2%)	0.742	1
SMARCA4	7 (5.1%)	1 (1.1%)	0.147	0.904
RBM10	8 (5.8%)	0 (0%)	0.023	0.35
RAD21	7 (5.1%)	1 (1.1%)	0.147	0.904
RET	4 (2.9%)	4 (4.3%)	0.718	1
MET	5 (3.6%)	2 (2.1%)	0.704	1
CCND1	6 (4.3%)	0 (0%)	0.084	0.647
CTNNB1	1 (0.7%)	5 (5.3%)	0.041	0.541
BRAF	6 (4.3%)	0 (0%)	0.084	0.647
U2AF1	6 (4.3%)	0 (0%)	0.084	0.647
FGF3	5 (3.6%)	1 (1.1%)	0.405	1
CDK4	6 (4.3%)	0 (0%)	0.084	0.647
GNAS	6 (4.3%)	0 (0%)	0.084	0.647
FGFR1	5 (3.6%)	0 (0%)	0.083	0.647
BRCA1	2 (1.4%)	3 (3.2%)	0.397	1
FGF19	4 (2.9%)	1 (1.1%)	0.651	1
SMAD4	4 (2.9%)	1 (1.1%)	0.651	1
RICTOR	5 (3.6%)	0 (0%)	0.083	0.647
TERT	4 (2.9%)	1 (1.1%)	0.651	1
C11orf30	4 (2.9%)	0 (0%)	0.149	0.904
APC	4 (2.9%)	0 (0%)	0.149	0.904
BRCA2	2 (1.4%)	2 (2.1%)	1	1
ARFRP1	4 (2.9%)	0 (0%)	0.149	0.904
NSD3	4 (2.9%)	0 (0%)	0.149	0.904
MUTYH	3 (2.2%)	1 (1.1%)	0.649	1
PDGFRA	2 (1.4%)	2 (2.1%)	1	1
NFE2L2	3 (2.2%)	1 (1.1%)	0.649	1
FGF10	3 (2.2%)	0 (0%)	0.274	1
NTRK1	2 (1.4%)	1 (1.1%)	1	1
KIT	2 (1.4%)	1 (1.1%)	1	1
PARK2	3 (2.2%)	0 (0%)	0.274	1
FGF4	2 (1.4%)	1 (1.1%)	1	1
ZNF217	3 (2.2%)	0 (0%)	0.274	1
PRKCI	3 (2.2%)	0 (0%)	0.274	1
NRAS	3 (2.2%)	0 (0%)	0.274	1
BCL2L2	3 (2.2%)	0 (0%)	0.274	1
SOX2	3 (2.2%)	0 (0%)	0.274	1
ASXL1	1 (0.7%)	2 (2.1%)	0.567	1
CBL	1 (0.7%)	2 (2.1%)	0.567	1
MCL1	3 (2.2%)	0 (0%)	0.274	1
KMT2D (MLL2)	1 (0.7%)	2 (2.1%)	0.567	1
TERC	3 (2.2%)	0 (0%)	0.274	1
MRE11A	3 (2.2%)	0 (0%)	0.274	1

JAK2	0 (0%)	3 (3.2%)	0.065	0.647
ROS1	1 (0.7%)	1 (1.1%)	1	1

Figure S3. Correlation of detected alterations with clinico-pathological features (top-20 mutated genes). The Y-axis indicates the percentage of patients with the altered genes across the X-axis for groups under study. **(A)** Smoking status (never-smoker = 153 and nnever-smoker = 68). **(B)** Histological diagnosis. Patients with other diagnoses were excluded from this analysis (nLUAD= 184 and nSCC = 29). **(C)** Sex (nmale = 122 and nfemale = 110). LUAD: adenocarcinoma. SCC: squamous cell carcinoma. *Adjusted p-value <0.05.

A

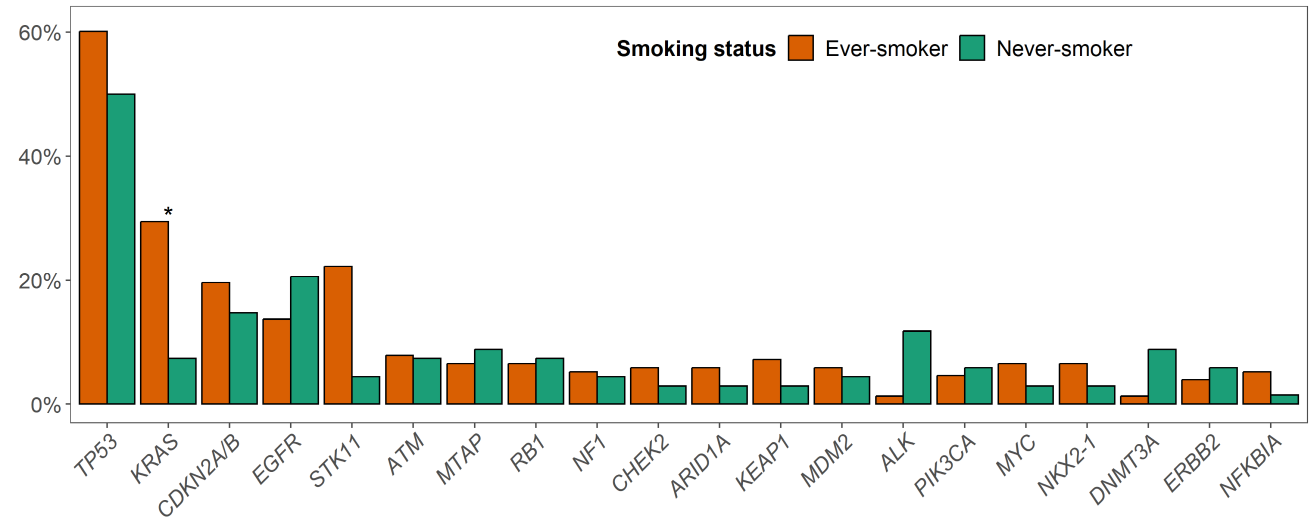

B

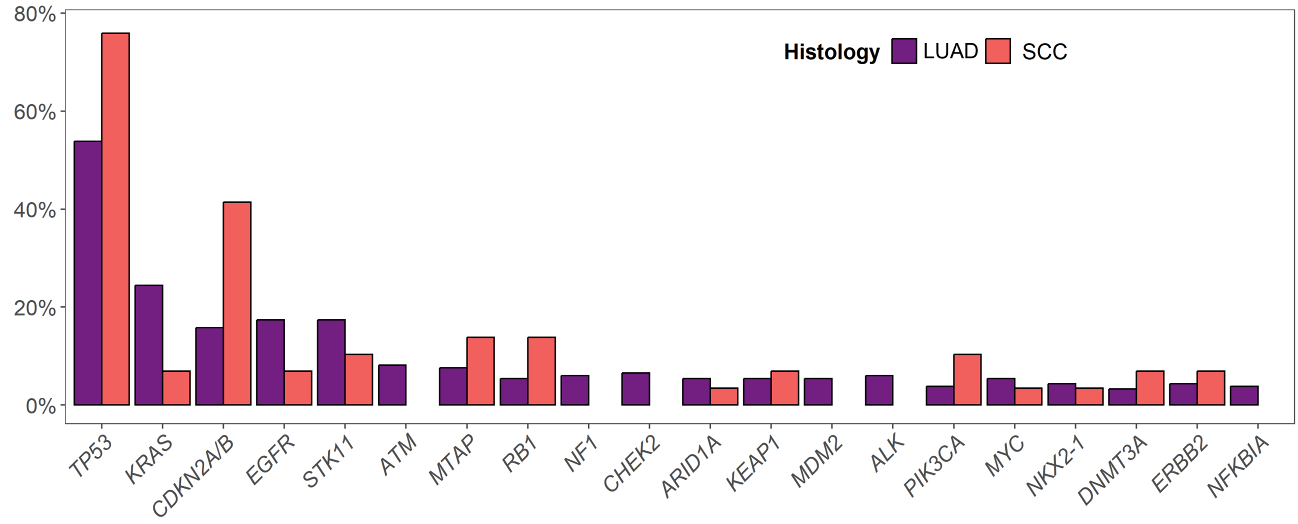

C

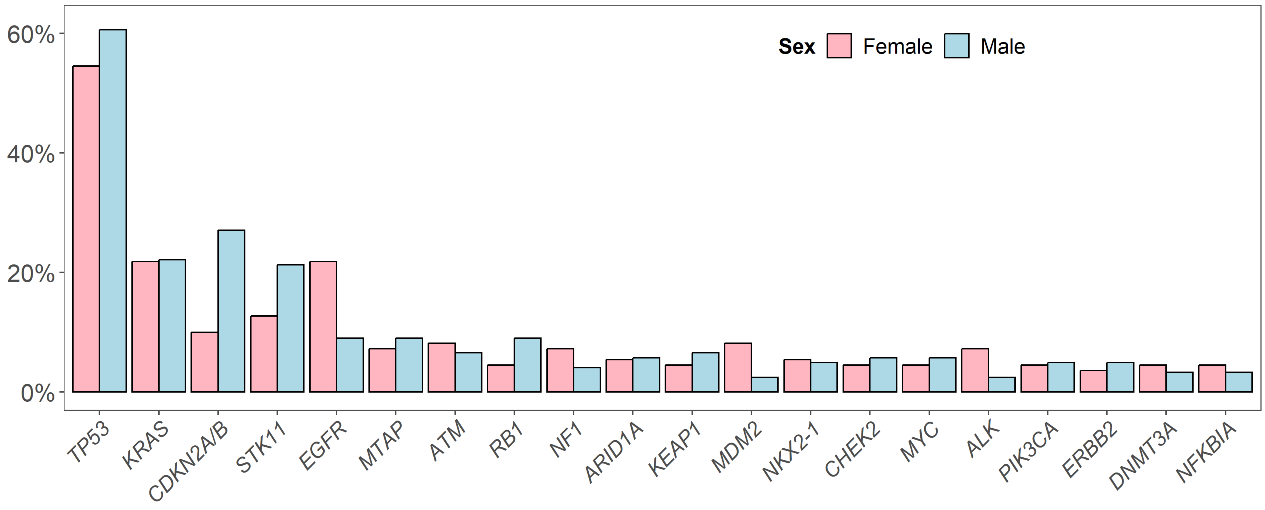

Table S2. Comparison of gene frequencies between ever-smokers and never-smokers. Selected genes altered in at least 1% of the cohort. Driver genes in NSCLC with frequency <1% were also included.

Gene	Ever-smoker (N=153)	Never-smoker (N=68)	P-value	Adjusted p-value
TP53	92 (60.1%)	34 (50%)	0.186	1
KRAS	45 (29.4%)	5 (7.4%)	0	0.034
CDKN2A/B	30 (19.6%)	10 (14.7%)	0.452	1
STK11	34 (22.2%)	3 (4.4%)	0.001	0.061
EGFR	21 (13.7%)	14 (20.6%)	0.232	1
MTAP	10 (6.5%)	6 (8.8%)	0.578	1
ATM	12 (7.8%)	5 (7.4%)	1	1
RB1	10 (6.5%)	5 (7.4%)	0.78	1
NF1	8 (5.2%)	3 (4.4%)	1	1
ARID1A	9 (5.9%)	2 (2.9%)	0.51	1
KEAP1	11 (7.2%)	2 (2.9%)	0.353	1
NKX2-1	10 (6.5%)	2 (2.9%)	0.352	1
CHEK2	9 (5.9%)	2 (2.9%)	0.51	1
MDM2	9 (5.9%)	3 (4.4%)	0.759	1
MYC	10 (6.5%)	2 (2.9%)	0.352	1
ALK	2 (1.3%)	8 (11.8%)	0.002	0.085
PIK3CA	7 (4.6%)	4 (5.9%)	0.741	1
ERBB2	6 (3.9%)	4 (5.9%)	0.501	1
TET2	5 (3.3%)	3 (4.4%)	0.704	1
PTEN	5 (3.3%)	3 (4.4%)	0.704	1
DNMT3A	2 (1.3%)	6 (8.8%)	0.012	0.489
NFKBIA	8 (5.2%)	1 (1.5%)	0.281	1
SMARCA4	7 (4.6%)	1 (1.5%)	0.44	1
RET	3 (2%)	5 (7.4%)	0.061	1
RBM10	8 (5.2%)	0 (0%)	0.11	1
RAD21	7 (4.6%)	1 (1.5%)	0.44	1
MET	3 (2%)	4 (5.9%)	0.206	1
CDK4	6 (3.9%)	0 (0%)	0.181	1
GNAS	5 (3.3%)	1 (1.5%)	0.669	1
FGF3	5 (3.3%)	0 (0%)	0.327	1

BRAF	4 (2.6%)	1 (1.5%)	1	1
CTNNB1	4 (2.6%)	2 (2.9%)	1	1
CCND1	5 (3.3%)	0 (0%)	0.327	1
U2AF1	5 (3.3%)	1 (1.5%)	0.669	1
BRCA1	3 (2%)	2 (2.9%)	0.644	1
RICTOR	5 (3.3%)	0 (0%)	0.327	1
SMAD4	4 (2.6%)	1 (1.5%)	1	1
FGF19	4 (2.6%)	0 (0%)	0.315	1
FGFR1	4 (2.6%)	0 (0%)	0.315	1
TERT	4 (2.6%)	0 (0%)	0.315	1
APC	2 (1.3%)	1 (1.5%)	1	1
PDGFRA	3 (2%)	0 (0%)	0.554	1
NSD3	3 (2%)	0 (0%)	0.554	1
MUTYH	4 (2.6%)	0 (0%)	0.315	1
BRCA2	3 (2%)	1 (1.5%)	1	1
NFE2L2	4 (2.6%)	0 (0%)	0.315	1
ARFRP1	3 (2%)	1 (1.5%)	1	1
C11orf30	3 (2%)	1 (1.5%)	1	1
TERC	2 (1.3%)	1 (1.5%)	1	1
ASXL1	3 (2%)	0 (0%)	0.554	1
KMT2D (MLL2)	1 (0.7%)	1 (1.5%)	0.522	1
MRE11A	3 (2%)	0 (0%)	0.554	1
MCL1	3 (2%)	0 (0%)	0.554	1
ZNF217	2 (1.3%)	1 (1.5%)	1	1
PARK2	2 (1.3%)	1 (1.5%)	1	1
JAK2	0 (0%)	3 (4.4%)	0.028	0.96
PRKCI	2 (1.3%)	1 (1.5%)	1	1
FGF10	3 (2%)	0 (0%)	0.554	1
BCL2L2	3 (2%)	0 (0%)	0.554	1
NRAS	3 (2%)	0 (0%)	0.554	1
SOX2	2 (1.3%)	0 (0%)	1	1
KIT	2 (1.3%)	0 (0%)	1	1
FGF4	2 (1.3%)	0 (0%)	1	1
NTRK1	3 (2%)	0 (0%)	0.554	1
CBL	2 (1.3%)	1 (1.5%)	1	1
ROS1	1 (0.7%)	1 (1.5%)	0.522	1

Table S3. Comparison of gene frequencies among histological subtypes. Patients with other diagnosis were excluded from this analysis. Selected genes altered in at least 1% of the cohort. Driver genes in NSCLC with frequency <1% were also included. LUAD: adenocarcinoma; SCC: squamous cell carcinoma.

Gene	LUAD (N=184)	SCC (N=29)	P-value	Adjusted p-value
TP53	99 (53.8%)	22 (75.9%)	0.028	0.854
KRAS	45 (24.5%)	2 (6.9%)	0.032	0.854
CDKN2A/B	29 (15.8%)	12 (41.4%)	0.004	0.318
STK11	32 (17.4%)	3 (10.3%)	0.429	1
EGFR	32 (17.4%)	2 (6.9%)	0.183	1
MTAP	14 (7.6%)	4 (13.8%)	0.279	1
ATM	15 (8.2%)	0 (0%)	0.232	1
RB1	10 (5.4%)	4 (13.8%)	0.105	0.854
NF1	11 (6%)	0 (0%)	0.368	1

ARID1A	10 (5.4%)	1 (3.4%)	1	1
KEAP1	10 (5.4%)	2 (6.9%)	0.67	1
NKX2-1	8 (4.3%)	1 (3.4%)	1	1
CHEK2	12 (6.5%)	0 (0%)	0.378	1
MDM2	10 (5.4%)	0 (0%)	0.364	1
MYC	10 (5.4%)	1 (3.4%)	1	1
ALK	11 (6%)	0 (0%)	0.368	1
PIK3CA	7 (3.8%)	3 (10.3%)	0.141	0.854
ERBB2	8 (4.3%)	2 (6.9%)	0.629	1
TET2	7 (3.8%)	0 (0%)	0.597	1
PTEN	5 (2.7%)	3 (10.3%)	0.079	0.854
DNMT3A	6 (3.3%)	2 (6.9%)	0.299	1
NFKBIA	7 (3.8%)	0 (0%)	0.597	1
SMARCA4	5 (2.7%)	2 (6.9%)	0.244	1
RET	8 (4.3%)	0 (0%)	0.602	1
RBM10	5 (2.7%)	1 (3.4%)	0.589	1
RAD21	6 (3.3%)	1 (3.4%)	1	1
MET	5 (2.7%)	0 (0%)	1	1
CDK4	5 (2.7%)	0 (0%)	1	1
GNAS	5 (2.7%)	0 (0%)	1	1
FGF3	4 (2.2%)	1 (3.4%)	0.523	1
BRAF	5 (2.7%)	1 (3.4%)	0.589	1
CTNNB1	4 (2.2%)	0 (0%)	1	1
CCND1	4 (2.2%)	1 (3.4%)	0.523	1
U2AF1	5 (2.7%)	1 (3.4%)	0.589	1
BRCA1	3 (1.6%)	2 (6.9%)	0.138	0.854
RICTOR	5 (2.7%)	0 (0%)	1	1
SMAD4	4 (2.2%)	1 (3.4%)	0.523	1
FGF19	3 (1.6%)	1 (3.4%)	0.446	1
FGFR1	2 (1.1%)	2 (6.9%)	0.09	0.854
TERT	5 (2.7%)	0 (0%)	1	1
APC	4 (2.2%)	0 (0%)	1	1
PDGFRA	4 (2.2%)	0 (0%)	1	1
NSD3	2 (1.1%)	1 (3.4%)	0.357	1
MUTYH	4 (2.2%)	0 (0%)	1	1
BRCA2	3 (1.6%)	1 (3.4%)	0.446	1
NFE2L2	0 (0%)	4 (13.8%)	0	0.048
ARFRP1	3 (1.6%)	0 (0%)	1	1
C11orf30	3 (1.6%)	1 (3.4%)	0.446	1
TERC	1 (0.5%)	2 (6.9%)	0.049	0.854
ASXL1	1 (0.5%)	1 (3.4%)	0.254	1
KMT2D (MLL2)	2 (1.1%)	1 (3.4%)	0.357	1
MRE11A	2 (1.1%)	0 (0%)	1	1
MCL1	3 (1.6%)	0 (0%)	1	1
ZNF217	3 (1.6%)	0 (0%)	1	1
PARK2	2 (1.1%)	1 (3.4%)	0.357	1
JAK2	3 (1.6%)	0 (0%)	1	1
PRKCI	1 (0.5%)	2 (6.9%)	0.049	0.854
FGF10	3 (1.6%)	0 (0%)	1	1
BCL2L2	1 (0.5%)	0 (0%)	1	1
NRAS	3 (1.6%)	0 (0%)	1	1
SOX2	0 (0%)	2 (6.9%)	0.018	0.764

KIT	2 (1.1%)	0 (0%)	1	1
FGF4	1 (0.5%)	1 (3.4%)	0.254	1
NTRK1	3 (1.6%)	0 (0%)	1	1
CBL	3 (1.6%)	0 (0%)	1	1
ROS1	2 (1.1%)	0 (0%)	1	1

Table S4. Comparison of gene frequencies between male and female. Selected genes altered in at least 1% of the cohort. Driver genes in NSCLC with frequency <1% were also included.

Gene	Male (N=122)	Female (N=110)	P-value	Adjusted p-value
TP53	74 (60.7%)	60 (54.5%)	0.355	1
KRAS	27 (22.1%)	24 (21.8%)	1	1
CDKN2A/B	33 (27%)	11 (10%)	0.001	0.215
STK11	26 (21.3%)	14 (12.7%)	0.117	1
EGFR	11 (9%)	24 (21.8%)	0.009	0.803
MTAP	11 (9%)	8 (7.3%)	0.811	1
ATM	8 (6.6%)	9 (8.2%)	0.802	1
RB1	11 (9%)	5 (4.5%)	0.204	1
NF1	5 (4.1%)	8 (7.3%)	0.394	1
ARID1A	7 (5.7%)	6 (5.5%)	1	1
KEAP1	8 (6.6%)	5 (4.5%)	0.577	1
NKX2-1	6 (4.9%)	6 (5.5%)	1	1
CHEK2	7 (5.7%)	5 (4.5%)	0.772	1
MDM2	3 (2.5%)	9 (8.2%)	0.073	1
MYC	7 (5.7%)	5 (4.5%)	0.772	1
ALK	3 (2.5%)	8 (7.3%)	0.122	1
PIK3CA	6 (4.9%)	5 (4.5%)	1	1
ERBB2	6 (4.9%)	4 (3.6%)	0.752	1
TET2	4 (3.3%)	5 (4.5%)	0.739	1
PTEN	6 (4.9%)	3 (2.7%)	0.505	1
DNMT3A	4 (3.3%)	5 (4.5%)	0.739	1
NFKBIA	4 (3.3%)	5 (4.5%)	0.739	1
SMARCA4	6 (4.9%)	2 (1.8%)	0.286	1
RET	2 (1.6%)	6 (5.5%)	0.154	1
RBM10	4 (3.3%)	4 (3.6%)	1	1
RAD21	4 (3.3%)	4 (3.6%)	1	1
MET	3 (2.5%)	4 (3.6%)	0.711	1
CDK4	1 (0.8%)	5 (4.5%)	0.104	1
GNAS	4 (3.3%)	2 (1.8%)	0.686	1
FGF3	2 (1.6%)	4 (3.6%)	0.426	1
BRAF	4 (3.3%)	2 (1.8%)	0.686	1
CTNNB1	2 (1.6%)	4 (3.6%)	0.426	1
CCND1	2 (1.6%)	4 (3.6%)	0.426	1
U2AF1	4 (3.3%)	2 (1.8%)	0.686	1
BRCA1	5 (4.1%)	0 (0%)	0.061	1
RICTOR	3 (2.5%)	2 (1.8%)	1	1
SMAD4	4 (3.3%)	1 (0.9%)	0.373	1
FGF19	2 (1.6%)	3 (2.7%)	0.67	1
FGFR1	4 (3.3%)	1 (0.9%)	0.373	1
TERT	2 (1.6%)	3 (2.7%)	0.67	1
APC	1 (0.8%)	3 (2.7%)	0.348	1

PDGFRA	1 (0.8%)	3 (2.7%)	0.348	1
NSD3	2 (1.6%)	2 (1.8%)	1	1
MUTYH	2 (1.6%)	2 (1.8%)	1	1
BRCA2	1 (0.8%)	3 (2.7%)	0.348	1
NFE2L2	3 (2.5%)	1 (0.9%)	0.624	1
ARFRP1	2 (1.6%)	2 (1.8%)	1	1
C11orf30	2 (1.6%)	2 (1.8%)	1	1
TERC	3 (2.5%)	0 (0%)	0.249	1
ASXL1	3 (2.5%)	0 (0%)	0.249	1
KMT2D (MLL2)	2 (1.6%)	1 (0.9%)	1	1
MRE11A	3 (2.5%)	0 (0%)	0.249	1
MCL1	3 (2.5%)	0 (0%)	0.249	1
ZNF217	1 (0.8%)	2 (1.8%)	0.605	1
PARK2	2 (1.6%)	1 (0.9%)	1	1
JAK2	2 (1.6%)	1 (0.9%)	1	1
PRKCI	3 (2.5%)	0 (0%)	0.249	1
FGF10	2 (1.6%)	1 (0.9%)	1	1
BCL2L2	2 (1.6%)	1 (0.9%)	1	1
NRAS	2 (1.6%)	1 (0.9%)	1	1
SOX2	3 (2.5%)	0 (0%)	0.249	1
KIT	1 (0.8%)	2 (1.8%)	0.605	1
FGF4	1 (0.8%)	2 (1.8%)	0.605	1
NTRK1	2 (1.6%)	1 (0.9%)	1	1
CBL	2 (1.6%)	1 (0.9%)	1	1
ROS1	1 (0.8%)	1 (0.9%)	1	1

Table S5. Driver genes alterations. N: number.

Gene	Variant	Tier	N	Proportion on gene groups	Proportion on total samples
KRAS	**G12C**	**I**	**21**	**36.8%**	**9.1%**
	non-G12C	-	16	28.1%	6.9%
	amplification	-	7	12.3%	3.0%
	Not available	-	3	5.3%	1.3%
	Q61H	-	3	5.3%	1.3%
	amplification-equivocal	-	2	3.5%	0.9%
	L19F	-	2	3.5%	0.9%
	Q61L	-	1	1.8%	0.4%
	A146V	-	1	1.8%	0.4%
	G13V	-	1	1.8%	0.4%
EGFR	**exon 19 deletion**	**I**	**15**	**34.1%**	**6.5%**
	Other	-	9	20.5%	3.4%
	**L858R**	**I**	**6**	**13.6%**	**2.6%**
	**exon 20 insertion**	**I**	**5**	**11.4%**	**2.2%**
	**T790M**	**I**	**4**	**9.1%**	**1.7%**
	amplification	-	3	6.8%	1.3%
	L861Q	-	2	4.5%	0.9%
ALK	**EML4-ALK fusion**	**I**	**9**	**69.2%**	**3.9%**
	G1202R	-	1	7.7%	0.4%
	G1128V	-	1	7.7%	0.4%
	**CLIP4-ALK fusion**	**I**	**1**	**7.7%**	**0.4%**

	**ALK-BIRC6 rearrangement**	**I**	**1**	**7.7%**	**0.4%**
ERBB2	**A775_G776insYVMA**	**II**	**3**	**25.0%**	**1.3%**
	amplification	-	3	25.0%	1.3%
	amplification-equivocal	-	2	16.7%	0.9%
	**S310F**	**II**	**1**	**8.3%**	**0.4%**
	**SNV not available**	**II**	**1**	**8.3%**	**0.4%**
	**780_Y781insGSP**	**II**	**1**	**8.3%**	**0.4%**
	**G776>LC**	**II**	**1**	**8.3%**	**0.4%**
RET	**KIF5B-RET fusion**	**I**	**7**	**77.8%**	**3.0%**
	**RET-ANK3 rearrangement**	**I**	**1**	**11.1%**	**0.4%**
	**CCDC6-RET fusion**	**I**	**1**	**11.1%**	**0.4%**
MET	**exon 14 splice site**	**I**	**3**	**42.9%**	**1.3%**
	**amplification**	**II**	**1**	**14.3%**	**0.4%**
	**H1094Y**	**I**	**1**	**14.3%**	**0.4%**
	**amplification-equivocal**	**II**	**1**	**14.3%**	**0.4%**
	**D1228N**	**I**	**1**	**14.3%**	**0.4%**
BRAF	**V600E**	**I**	**2**	**33.3%**	**0.9%**
	G466V	-	1	16.7%	0.4%
	N581I	-	1	16.7%	0.4%
	G469V	-	1	16.7%	0.4%
	K601E	-	1	16.7%	0.4%
NTRK1	**SQSTM1-NTRK1 fusion**	**I**	**1**	**25.0%**	**0.4%**
	**rearrangement exon 8**	**I**	**1**	**25.0%**	**0.4%**
	amplification-equivocal	-	1	25.0%	0.4%
	**EPS15-NTRK1 fusion**	**I**	**1**	**25.0%**	**0.4%**
ROS1	**LRIG3-ROS1 fusion**	**I**	**1**	**50.0%**	**0.4%**
	**CD74-ROS1 fusion**	**I**	**1**	**50.0%**	**0.4%**

Figure S4. TMB by histology (n_{LUAD} = 108, n_{SCC} = 23, and n_{other} = 11). TMB: tumor mutational burden; LUAD: adenocarcinoma; SCC: squamous cell carcinoma.

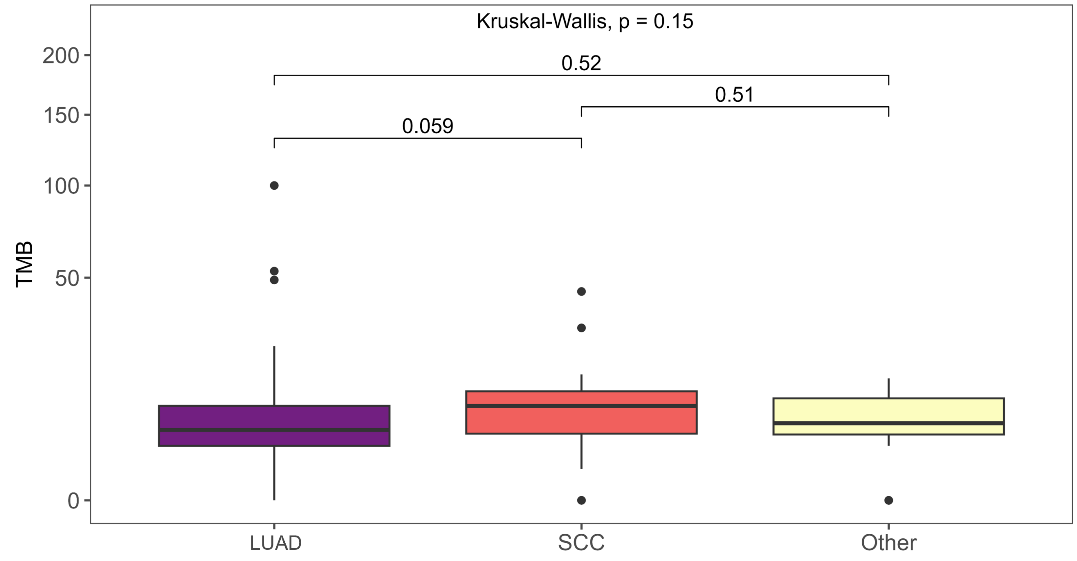

Figure S5. Microsatellite status: tissue-based (n=138) vs ctDNA (n=94) NGS. MS: microsatellite status.

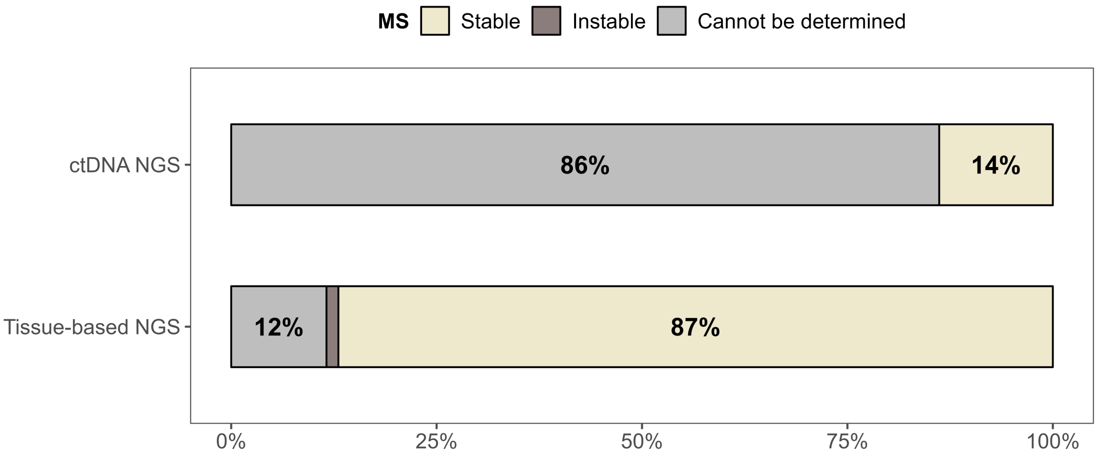

Figure S6. Distribution of co-occurring alterations (in driver genes or with n>1) in patients with altered (**A**) *KRAS*, (**B**) *EGFR*, (**C**) *ERBB2*, (**D**) *ALK*, (**E**) *RET*, (**F**) *MET*, and (**G**) *BRAF*.

A. ***KRAS*** **(N=51)**

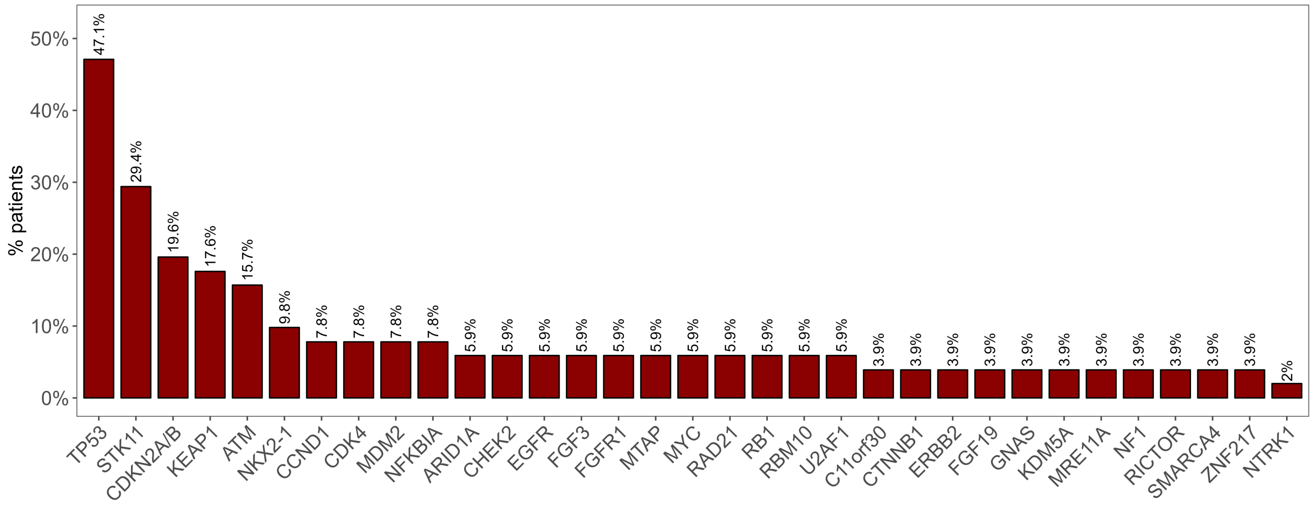

B. ***EGFR*** **(N=35)**

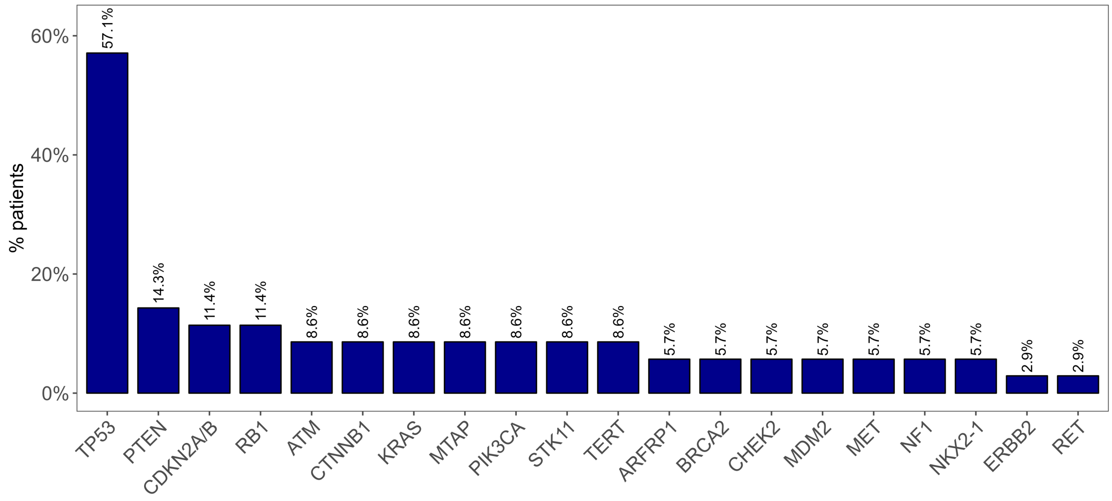

C. ***ERBB2*** **(N=10)**

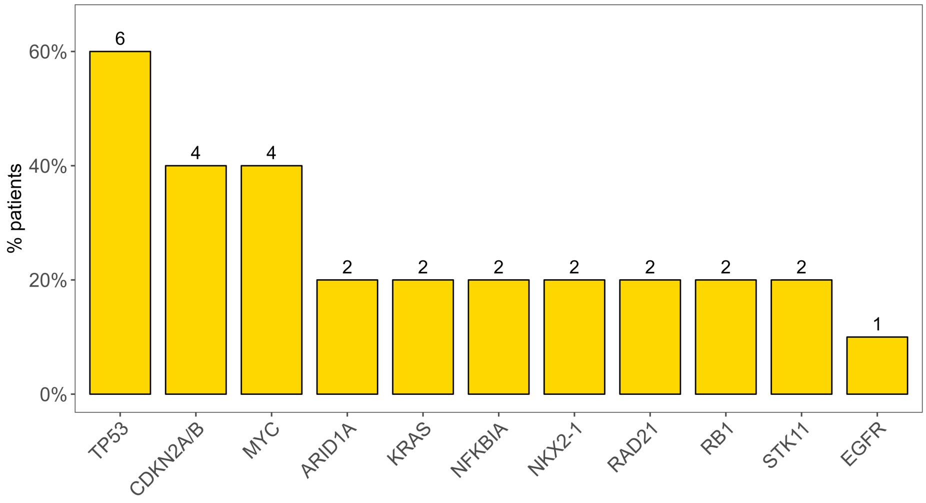

D. ***ALK*** **(N=11)**

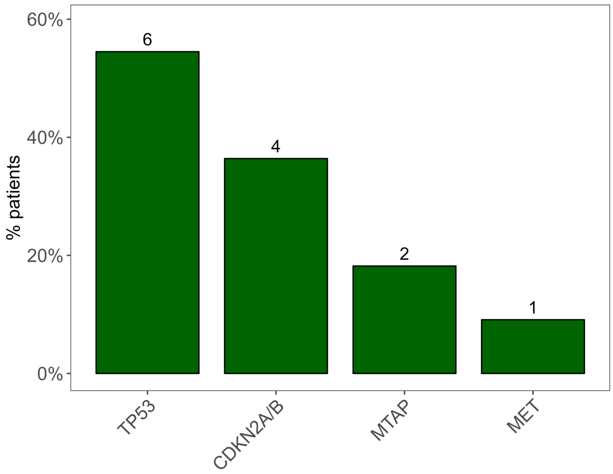

E. ***RET*** **(N=8)**

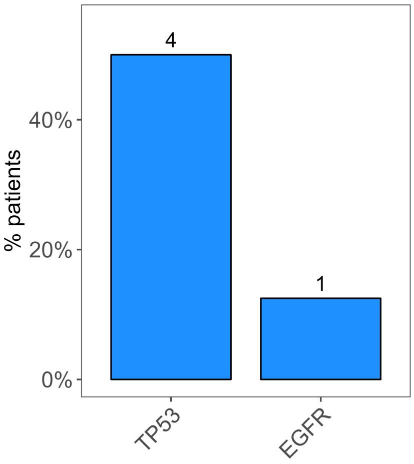

F. ***MET*** **(N=7)**

G. ***BRAF*** **(N=6)**

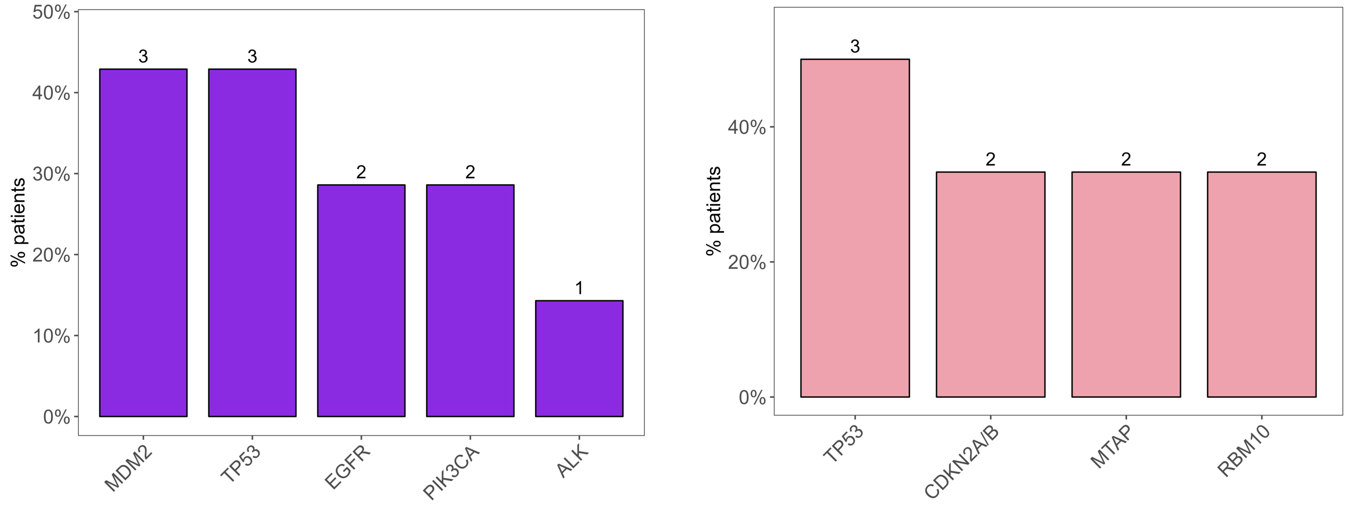

Figure S7. Co-occurrence and mutual exclusivity of top-20 genes/driver genes in (**A**) tissue (n = 138) and (**B**) liquid biopsy samples (n = 94). P-values (not-adjusted) were calculated using Fisher's exact test and transformed into a score, -log10(P-value). * Adjusted p-value <0.05.

A **B**

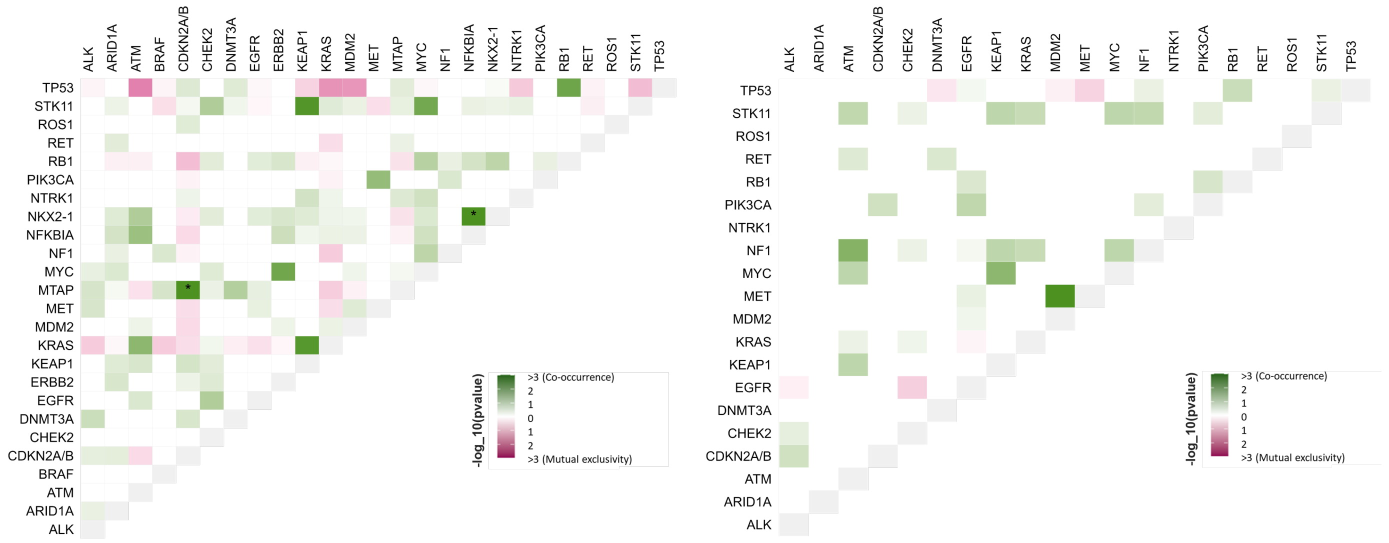

Figure S8. Kaplan-Meier Curves indicating overall survival from histological and metastatic diagnosis stratified by (**A**, **B**) stage, (**C**, **D**) ECOG performance status, (**E**, **F**) smoking status, and (**G**) number of altered genes per sample. ECOG: Eastern Cooperative Oncology Group; OS: overall survival; PS: performance status.

OS from histological diagnosis **OS from metastatic diagnosis**

A **B**

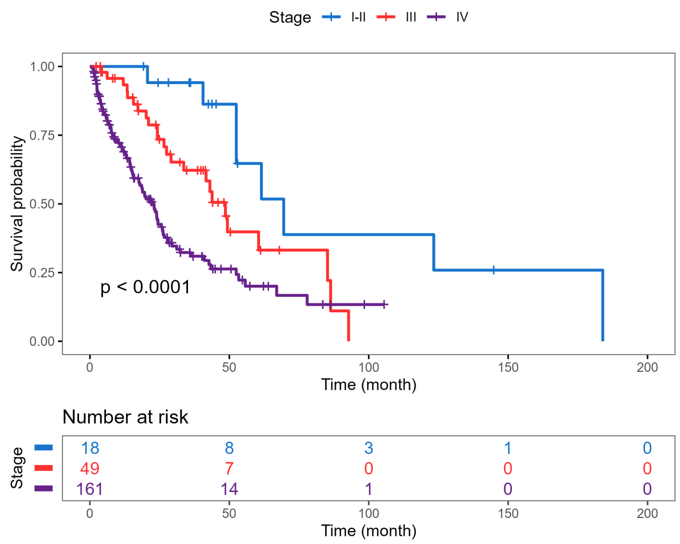
Stage I-II III IV
Survival probability
p < 0.0001
Time (month)
Number at risk
Stage
18 8 3 1 0
49 7 0 0 0
161 14 1 0 0

C

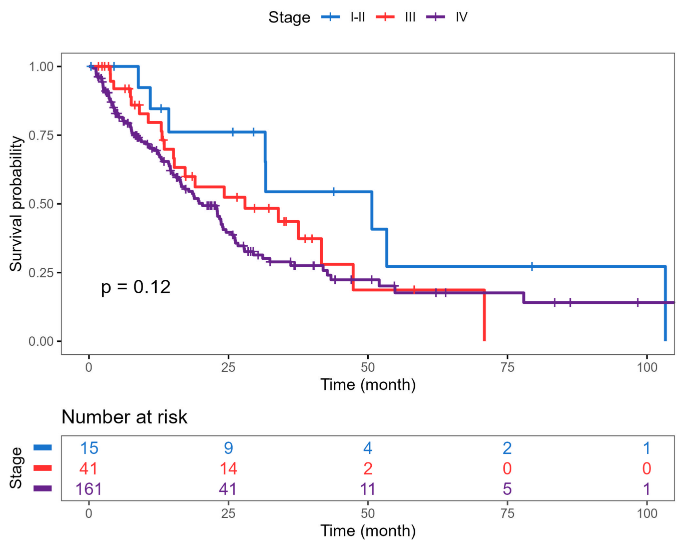
Stage I-II III IV
Survival probability
p = 0.12
Time (month)
Number at risk
Stage
15 9 4 2 1
41 14 2 0 0
161 41 11 5 1

D

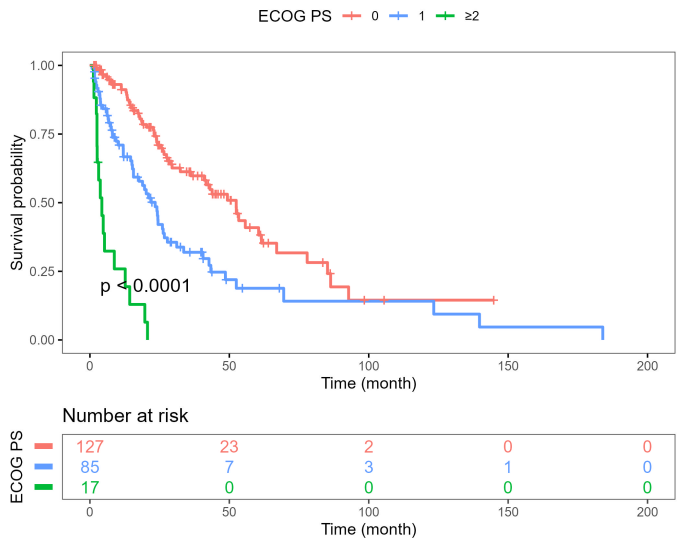
ECOG PS 0 1 ≥2
Survival probability
p < 0.0001
Time (month)
Number at risk
ECOG PS
127 23 2 0 0
85 7 3 1 0
17 0 0 0 0

E

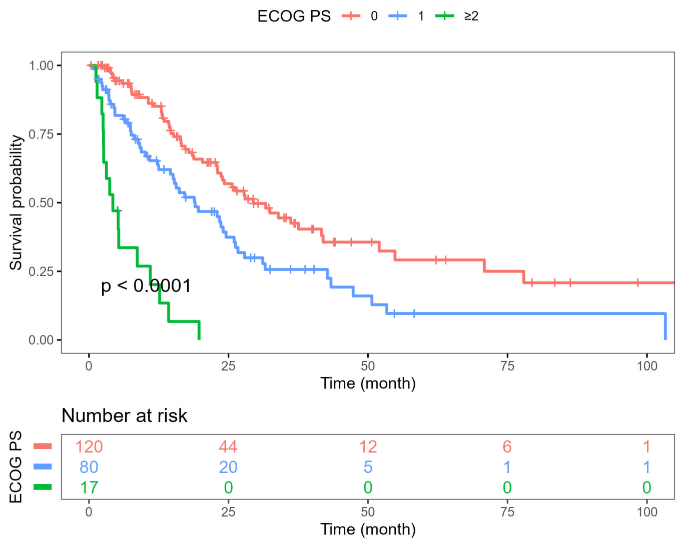
ECOG PS 0 1 ≥2
Survival probability
p < 0.0001
Time (month)
Number at risk
ECOG PS
120 44 12 6 1
80 20 5 1 1
17 0 0 0 0

F

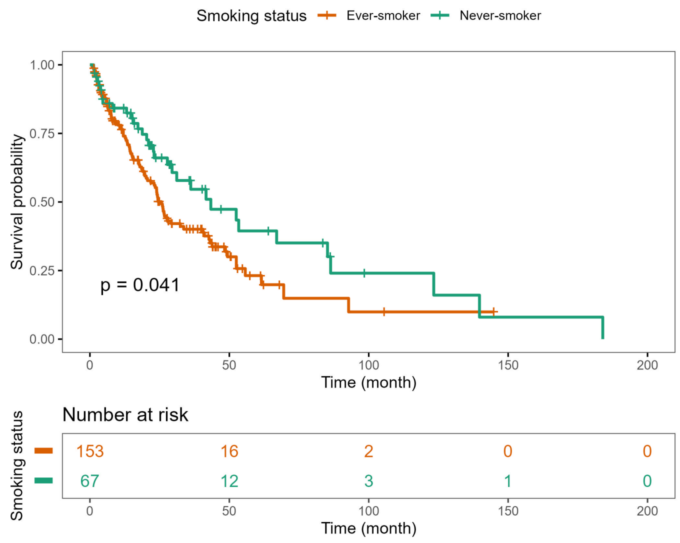
Smoking status Ever-smoker Never-smoker
Survival probability
p = 0.041
Time (month)
Number at risk
Smoking status
153 16 2 0 0
67 12 3 1 0

G

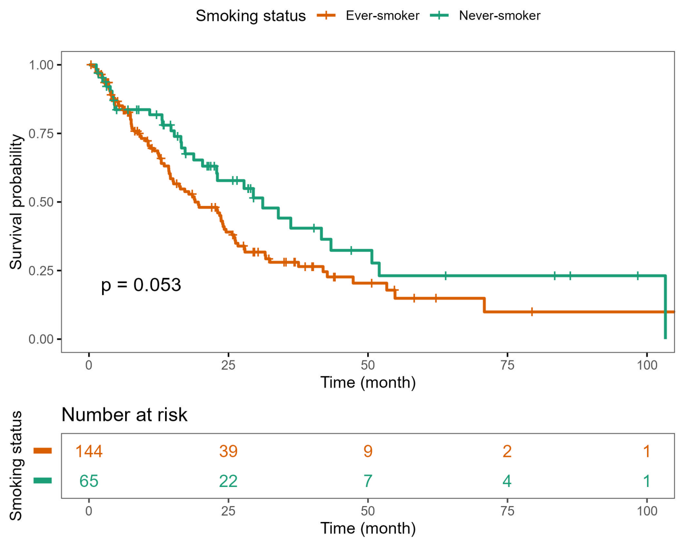
Smoking status Ever-smoker Never-smoker
Survival probability
p = 0.053
Time (month)
Number at risk
Smoking status
144 39 9 2 1
65 22 7 4 1

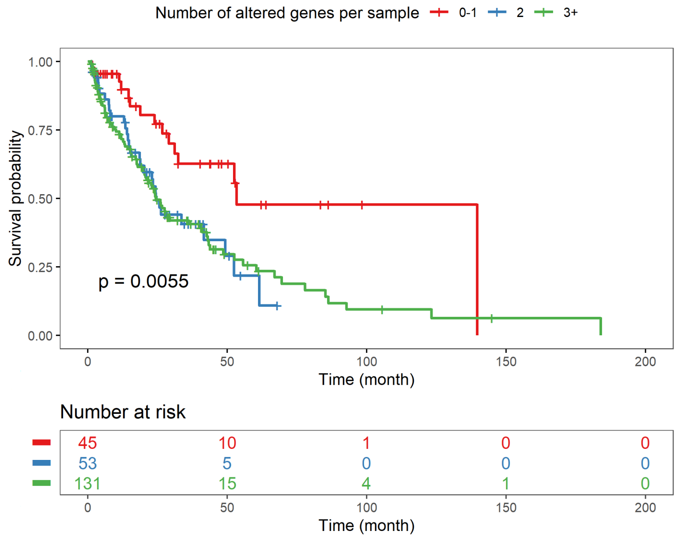

Figure S9. One-year conditional mOS by NGS timing. The x-axis represents the duration of survival to date (6-month intervals). All patients in the "Diagnosis" group died within 42 months; therefore, they were assumed to have a constant survival rate from that point onwards.

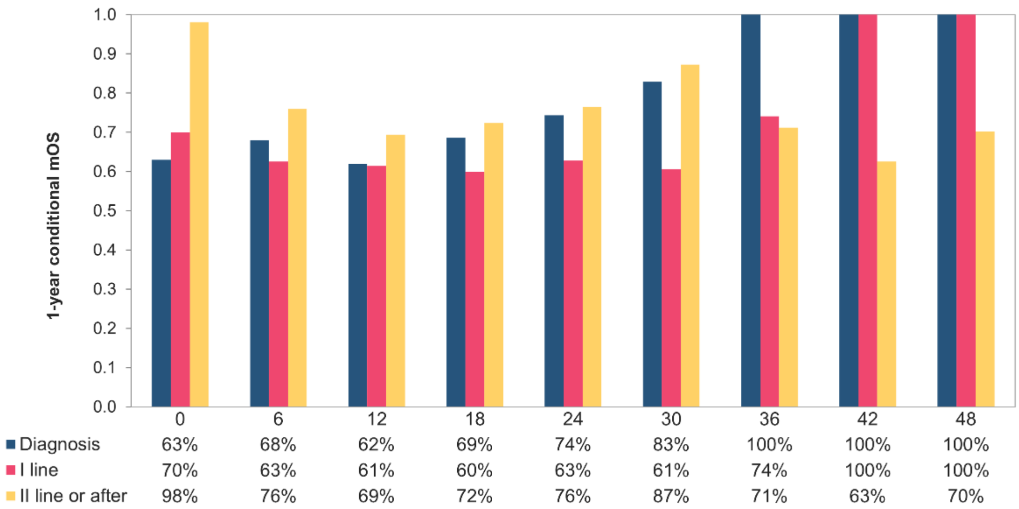

Supplement: Supplementary file 1 [file DataSheet_1.pdf]
